# Secondary and primary metabolites reveal putative resistance-associated biomarkers against *Erysiphe necator* in resistant grapevine genotypes

**DOI:** 10.3389/fpls.2023.1112157

**Published:** 2023-01-31

**Authors:** Ramona Mihaela Ciubotaru, Pietro Franceschi, Silvia Vezzulli, Luca Zulini, Marco Stefanini, Michael Oberhuber, Peter Robatscher, Giulia Chitarrini, Urska Vrhovsek

**Affiliations:** ^1^ Department of Agri-Food, Environmental and Animal Sciences, University of Udine, Udine, Italy; ^2^ Food Quality and Nutrition Department, Research and Innovation Centre, Fondazione Edmund Mach, San Michele all’Adige, Italy; ^3^ Unit of Computational Biology, Research and Innovation Centre, Fondazione Edmund Mach, San Michelle All'Adige, Italy; ^4^ Genomics and Biology of Fruit Crops Department, Research and Innovation Centre, Fondazione Edmund Mach, San Michelle All'Adige, Italy; ^5^ Laboratory for Flavours and Metabolites, Laimburg Research Centre, Auer (Ora), Italy

**Keywords:** powdery mildew, metabolomics, GC-MS, LC-MS, resistance, loci, biomarkers

## Abstract

Numerous fungicide applications are required to control *Erysiphe necator*, the causative agent of powdery mildew. This increased demand for cultivars with strong and long-lasting field resistance to diseases and pests. In comparison to the susceptible cultivar ‘Teroldego’, the current study provides information on some promising disease-resistant varieties (mono-locus) carrying one *E. necator-*resistant locus: BC4 and ‘Kishmish vatkana’, as well as resistant genotypes carrying several *E. necator* resistant loci (pyramided): ‘Bianca’, F26P92, F13P71, and NY42. A clear picture of the metabolites’ alterations in response to the pathogen is shown by profiling the main and secondary metabolism: primary compounds and lipids; volatile organic compounds and phenolic compounds at 0, 12, and 48 hours after pathogen inoculation. We identified several compounds whose metabolic modulation indicated that resistant plants initiate defense upon pathogen inoculation, which, while similar to the susceptible genotype in some cases, did not imply that the plants were not resistant, but rather that their resistance was modulated at different percentages of metabolite accumulation and with different effect sizes. As a result, we discovered ten up-accumulated metabolites that distinguished resistant from susceptible varieties in response to powdery mildew inoculation, three of which have already been proposed as resistance biomarkers due to their role in activating the plant defense response.

## 1 Introduction

*Vitis* is a genus widely dispersed and with diverse taxonomy, yet practically most of the world’s commercial grape production is focused on a single species, *Vitis vinifera* L., which is native to Europe and Asia Minor. *Vitis vinifera* is a species highly susceptible to various economically devastating pests and diseases, such as powdery mildew. This disease has several causal agents depending on the plant host. In grapevine, the causal agent of powdery mildew is the ascomycete *E. necator* [(syn. *Uncinula necator* (Schweinf.) Burrill] (Gadoury et al., 2012; Dry and Thomas, 2015).

Originating from northern America, grapevine powdery mildew was recently discovered in extremely diverse climatic conditions, including temperate regions with high rainfall, especially during spring months (Pirrello et al., 2019). The causal pathogen is obligatorily parasitic on the genus *Vitis*, as well as on *Cissus*, *Parthenocissus*, and *Ampelopsis* within the Vitaceae family (Gadoury et al., 2012). *Erysiphe necator* can infect all green tissues of the host and cause significant losses in yield and reduction in berry quality (Pimentel et al., 2021). Due to the devastating effects of the disease, breeding studies have been initiated to develop varieties that are tolerant or resistant to this disease all over the world (Atak and Şen, 2021; Atak, 2022).

During the infection process, *E. necator* produces conidia that germinate and grow epiphytically on the plant tissue forming a germ tube and a lobed appressorium. This breaks the cell wall invading the underlying epidermal cells with haustoria, a feeding structure. Through it, the fungus retrieves nutrients and secretes effectors that suppress the plant’s immunity, PAMP (pathogen-associated molecular pattern)-triggered immunity (PTI), allowing the colonization of plant tissue surfaces by the development of secondary hypha. The newly formed conidiophores sporulate to infect other host tissues and start a new infection cycle, which leads to an effector-triggered susceptibility (ETS) within the host (Gadoury et al., 2012). As an answer, the plants react using resistance (R) genes that are related to their evolutionary history (Feechan et al., 2011). These genes encode mainly for NBS-LRR (nucleotide-binding site – leucine-rich repeat) proteins that regularly express an interaction of the effector-triggered immunity (ETI) type in which the NB-LRR proteins act as receptors interacting with the strain-specific effectors of the pathogen released during infection. This is likewise true for the R genes that are transcribed into the *Vitacea*e plant family after *E. necator* infection (Qiu et al., 2015). The interaction generates a signaling cascade that leads to transcriptional re-programming in the host plant. The R genes activate several defense responses, including programmed cell death, the generation of reactive oxygen species, biosynthesis/signaling of plant stress/defense hormones, phytoalexin biosynthesis, and cell wall strengthening (Agurto et al., 2017; Welter et al., 2017).

Powdery mildew threatens many commercially important grapevine species and varieties, and thus, nowadays, the most used and efficient method of control is based on chemical treatments (Dry and Thomas, 2015). The most suitable fungicides against *E. necator* are benzimidazoles, ergosterol biosynthesis inhibitors, the quinone-outside inhibitor (QoI) compounds (strobilurins, quinolones), and the succinate dehydrogenase inhibitor (SDHI) group. Since the majority of these fungicides are site-specific, their repeated use leads to fungicide-resistant isolates (Gadoury et al., 2012). Thus, the introduction of resistant cultivars represents the most promising strategy to reduce the use of fungicides in viticulture, avoiding the appearance of *E. necator* resistance isolates. Although all *V. vinifera* cultivars are highly susceptible to *E. necator*, several *Vitaceae* species belonging to various American and Asian genotypes have developed resistance mechanisms against this pathogen (Gadoury et al., 2012; Agurto et al., 2017; Schneider et al., 2019). The resistance quantitative trait loci (QTLs) in *Vitaceae* are clustered in tandem repeats of genomic areas that have been genetically mapped, revealing many loci that encode R gene sequences conferring resistance on *E. necator* and have been utilized to obtain resistant plants by pseudo-backcrossing (Agurto et al., 2017). The R genes identified in *Vitaceae* are named Ren (i.e. resistance to *E. necator*) and Run (i.e. resistance to *Uncinula necator*).

To date, 17 grapevine powdery mildew resistance loci have been identified and described (Sosa-Zuniga et al., 2022); a descriptive list of them is available online (www.vivc.de/loci). It is important to note, however, that the presence of only one gene or locus, even if it has a large effect, can favor the selection of fungus isolates capable of overcoming resistance (McDonald and Linde, 2002). In other words, if the resistance is based solely on the presence of a gene, the fungus may mutate and evade immune recognition through the emergence of new virulent isolates.

In this context, better and longer-lasting disease resistance would be beneficial (Merdinoglu et al., 2018) and a pyramiding technique that integrates multiple resistance loci in the same genotype has been proposed (Mundt, 2018) as a potential solution. To guarantee the longevity of this type of resistance, it is required that loci with different mechanisms of action, spectrums of target isolates and contributions (minor and major) to the resistance be combined. This approach should bring in a more improved, durable and secure implementation strategy, given that, if any mutation or virulence factor occurs, the pathogen will be still recognized by at least one R gene (Peressotti et al., 2010; Cadle-Davidson et al., 2011; Feechan et al., 2015; Pap et al., 2016; Agurto et al., 2017).

Understanding disease resistance or tolerance mechanisms against *E. necator* in grapevine cultivars with different resistant loci at various time points post-inoculation may provide a holistic interpretation of the incompatible interactions between *Vitis* and *E. necator* and provide valuable information for breeding programs. In this respect, characterizing the metabolic profiles associated with disease resistance and susceptibility represents a key step for the identification of trait-related biomarkers. As we have seen in our previous study (Ciubotaru et al., 2021), metabolomics provided novel insights into the resistance mechanisms underlying the hybrid-pathogen interaction by identifying 22 putative biomarkers of grapevine resistance to *Plasmopara viticola.* Thus, the aim of our study is to provide important metabolomics evidence by monitoring changes in the concentration of a large set of metabolites belonging to four chemical classes in grapevine leaves subjected to artificial infection with *E. necator*. The significance of these findings is important for experiments studying the different behavior of resistant (totally or partially) varieties and susceptible ones in terms of the biochemical mechanisms involved in disease resistance. A better understanding of resistance biochemistry may lead to an improved selection of resistant plants promoting the reduction of fungicide treatments.

In this sense, metabolomics provides a comprehensive and quantitative investigation of metabolites belonging to both primary and secondary classes, including metabolites that play an important role in fighting pathogens. Moreover, metabolomics studies can help in the identification of key metabolites in plant adaptation to biotic stress. Despite the broad interest in more sustainable agriculture, metabolomics studies performed so far have focused on understanding the mechanism of grapevine defense against downy mildew, while only a limited number of investigations focused on *E. necator* (Pimentel et al., 2021). Recent studies have shown the mechanisms underlying the synergy between metabolomics and various omics approaches (Maia et al., 2020; Pimentel et al., 2021; Sosa-Zuniga et al., 2022; Yin et al., 2022), the metabolic differences in the composition of the berries and leaves in several grapevine cultivars (Atak et al., 2021; Rienth et al., 2021) as well as control of the pathogen (Gur et al., 2022).

In this work, we focused on two mono-locus resistant genotypes (‘VRH 3082-1-42’- commonly named BC4 - and ‘Kishmish vatkana’) and four pyramided resistant genotypes (‘Bianca’, F29P92, F13P71, and NY42) comparing them with the susceptible cultivar (cv) ‘Teroldego’. To date, our current work is the first study that addresses the way *E. necator* induces metabolic changes in grapevine genotypes harboring one or more R loci.

## 2 Material and methods

### 2.1 Genetic material

Six different resistant grapevine genotypes and the *V. vinifera* cv ‘Teroldego’ which is highly susceptible to powdery mildew were used in this study. BC4 and ‘Kishmish vatkana’ had a mono-locus resistance to powdery mildew, whereas ‘Bianca’, F26P92, F13P71, and NY42 had a pyramided resistance. The grapevine varieties, their pedigree, and their resistance-related loci are listed in **Table 1**.

**Table 1:** Grapevine varieties used in this study together with their origin [^1^ North American *Vitis*; ^2^ Asian *Vitis*; ^3^ Interspecific hybrids of *V. vinifera* with North American *Vitis species*, ^4^ pure *V. vinifera*], host response [PCD (programmed cell death), ROSs (reactive oxygen species)] and their powdery mildew associated resistance related loci *(Ren/Run)*.

Genotypes		Resistance related powdery mildew loci (Ren/Run)	Resistance mechanism within the hosts			Preliminary leaf resistance level	Source of resistance	References
			PCD	ROS	Callose			
mono-locus resistance	BC4	*Run1*	yes	yes	yes	total resistance	*M. rotundifolia* ^1^	Feechan et al., 2013; Agurto et al., 2017;
	‘Kishmish vatkana’	*Ren1*	yes	yes	yes	partial resistance	*V. vinifera* ^4^	Hoffmann et al., 2008;
pyramided resistance	‘Bianca’	*Ren3*	yes	yes	yes	partial resistance	*V. rupestris* ^3^	Welter et al., 2007; Zendler et al., 2020;
		*Ren9*	yes	n.d	n.d	partial resistance	*V. rupestris* ^3^	Zendler et al., 2017; Zendler et al., 2020;
	F26P92	*Ren3*	yes	yes	yes	partial resistance	*V. rupestris* ^3^	Welter et al., 2007; Zendler et al., 2020;
		*Ren9*	yes	n.d	n.d	partial resistance	*V. rupestris* ^3^	Zendler et al., 2017; Zendler et al., 2020;
	F13P71	*Run1*	yes	yes	yes	total resistance	*M. rotundifolia* ^1^	Feechan et al., 2013; Agurto et al., 2017;
		*Ren1*	yes	yes	yes	partial resistance	*V. vinifera* ^2^	Hoffmann et al., 2008;
	NY42	*Run1*	yes	yes	yes	total resistance	*M. rotundifolia* ^1^	Feechan et al., 2013; Agurto et al., 2017;
		*Ren2*	yes	n.d	n.d	partial resistance	*V. cinerea* ^2^	Feechan et al., 2015;
		*Ren3*	yes	yes	yes	partial resistance	*V. rupestris* ^3^	Welter et al., 2007; Zendler et al., 2020;
		*Ren9*	yes	n.d	n.d	partial resistance	*V. rupestris* ^3^	Zendler et al., 2017; Zendler et al., 2020;
control	‘Teroldego’	*-*	*-*	*-*	*-*	susceptible	*-*	

The levels of resistance described in the table: Total = greatly suppressed symptoms or the absence of visible symptoms; Partial = in cases where the symptomatology decreases without disappearing completely (Sosa-Zuniga et al., 2022; Julius Kühn-Institut, 2022).

The so-called BC4 hybrid was created in France and was derived from the intergeneric cross between *Muscadinia rotundifolia* and *V. vinifera* (Volynkin et al., 2021). It is resistant to the pathogen *E. necator* through the locus *Run1*, which is the earliest *E. necator* resistance loci to be identified in grapevine and one of the very few well characterized from the causal gene viewpoint (Agurto et al., 2017). The genotype ‘Kishmish vatkana’ is a cultivated grape from Central Asia obtained from the cross of ‘Vasarga chernaya’ with ‘Sultanina’ and resistant trough *Ren1* locus (Hoffmann et al., 2008).

‘Bianca’ is a hybrid between ‘Bouvier’ and ‘Villard Blanc’ created in 1963 at the Kölyuktetö - viticulture research facility in Hungary. Its resistance is conferred by the *Ren3* locus that was discovered on chromosome 15 of the hybrid ‘Regent’ (Welter et al., 2007) and the *Ren9* locus.

F29P92 and F13P71 are two pyramided hybrids created at Fondazione Edmund Mach (Italy). F26P92 is a mid-resistant genotype derived from ‘Bianca’ and ‘Nosiola’ with two resistance loci, *Ren3* and *Ren9*, while F13P71 is a cross between BC4 and ‘Kishmish vatkana’ having resistance through *Run1* and *Ren1* loci. The pyramided genotype NY42 is derived from a cross performed at USDA-Geneva (NY-USA) between NY95 and Eger99 and its resistance is given by the loci *Run1*, *Ren2*, *Ren3*, and *Ren9*. All three pyramided genotypes are considered breeding selections as they are still under the evaluation process. As a result, our paper is the first to report them in the literature.

The genotypes were grafted on Kober 5BB rootstock and grown in potted soil in controlled greenhouse conditions at the Fondazione Edmund Mach located in San Michele all’Adige (Trento), Italy (46^0^ 12′ 0′′ N, 11^0^ 8′ 0′′ E). Fourteen days prior to the experiment, all plants were treated with sulfur to make sure they were uniformly healthy. The sulfur treatment was repeated at the beginning of the experiment for all non-inoculated plants, which represented the control.

### 2.2 Pathogen inoculation

The inoculation of *E. necator* onto grapevine-potted plants in the greenhouse was done using conidia from the greenhouse and field; thus, the inoculum actually represented a mixture of *E. necator* strains.

The pathogen requires strict and stable climatic conditions for proper development, which is why in this study we tested two inoculation methods by following three different protocols. Three to four infected leaves were used as a source of inoculum for each round of inoculation depending on the spore quantity present on the leaves.

#### 2.2.1 Dry inoculation

**Figure 1:**
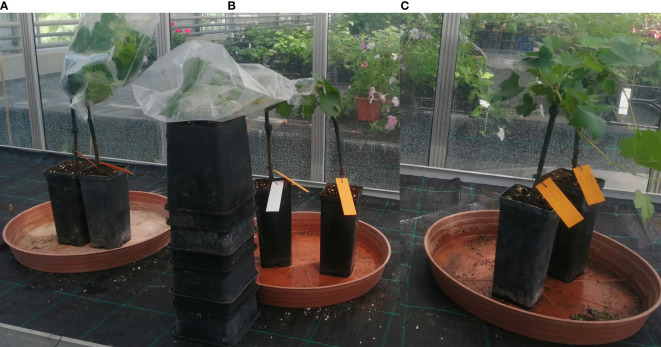
The artificial inoculation of *E. necator* conidia onto a susceptible genotype using three different methods: **(A)** - dry dispersion of spores covered by a stapled funnel (left); **(B)** - spray of a conidial suspension covered with plastic (middle); **(C)** - spray of a conidial suspension air-dried (right).

The first inoculation method was a dry dispersion of spores. For this method, we tested a combination of Deliere et al. (2010) modified protocol: the upper surfaces of healthy leaves were inoculated by dispersing spores with an air pump from infected leaves, and a cellophane funnel as per Valdés-Gómez et al. (2011) was placed around the inoculated shoots. Funnels were stapled, to allow air circulation, and were left in place for 24 h instead of 12 h as per the original inoculation method of Deliere et al. (2010) (**Figure 1A**-left).

#### 2.2.2 Wet inoculation

The second method of pathogen inoculation was based on a conidial suspension. We tested the protocol described by Atak (2017). We collected conidia of *E.necator* by washing three severely infected grapevine leaves in 15 ml of sterile distilled water with one drop of Tween-20 (2µl). The conidial suspension obtained had a concentration of 8.4 at 10^5^ conidia mL^-1^. Leaves were inoculated by spraying the conidia suspension using a spraying bottle of 10 mL, using roughly 0.5 mL of suspension per leaf (4 times spray per leaf). Inoculated leaves were immediately covered with thin plastic for 24 hours to obtain high humidity (**Figure 1B**-middle). For the same method (conidial suspension), we also tested the protocol described by Miclot et al. (2012) in which the above-prepared suspension was used to spray the upper surface of the leaves. The plants were subsequently air-dried using a ventilator and left uncovered (**Figure 1C**-right).

We carried out our experiment using the dry inoculation method. For each individual plant, the second, third and fourth fully expanded leaves from below the apex were inoculated by dusting the spores with an air pump for aquariums Newa Wind (Newa Tecno Industria, IT) that had attached a Pasteur glass. The spores were dusted directly into the adaxial surface of the leaves. The climatic conditions in the greenhouse were set at min 20°C – max of 22°C for temperature and 80% for relative humidity (Pertot and Gessler, 2006).

To evaluate the success of the experiments and of the inoculation with *E.necator*, we measured a parameter related to the pathogen performance: the OIV - 455 descriptor at 3, 7, and 11 dpi according to Miclot et al. (2012) (**Supplementary Table 1**). Briefly, we monitored the disease progression on a daily basis and quantified it based on observations of the plants’ reactions.

### 2.3 Experimental design

**Figure 2:**
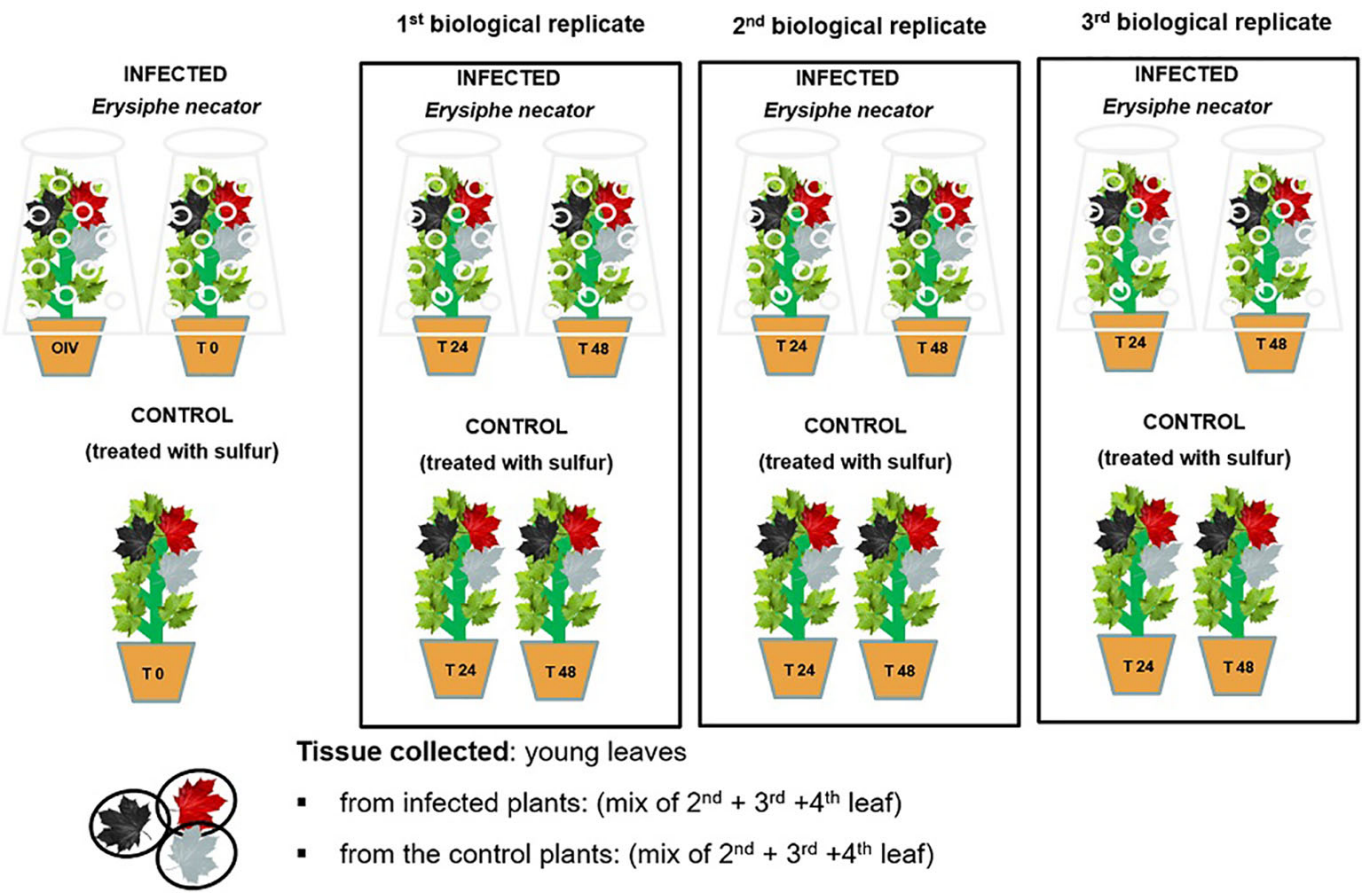
A schematic representation of the randomized experimental design of *E. necator’s* inoculation.

Around the twelve-leaf shoot stage, the plants (n=15 plants/genotype) were randomly sorted into two homogenous groups: control and inoculated. The two groups were kept in the same greenhouse (under same conditions) separated by a physical barrier to create two separate compartments in order to prevent any possible transmission of the pathogen. The plant material (three leaves below the shoot apex) was collected at 0, 24, and 48 h post-inoculation (hpi), starting from the morning (8:00 am, which is time zero), and immediately stored at -80°C until use. Three biological replicates were performed per time-point (**Figure 2**). The experiment was conducted for a 2-year period, in 2019 and 2021.

### 2.4 Metabolomics analysis

Extraction procedure and analysis of compounds:

Primary compounds were extracted following the method published by Chitarrini et al. (2017a). They were then subjected to derivatization using methoxamine hydrochloride in pyridine and later N-methyl-N-trimethylsilyl-trifluoroacetamide with 1% trimethylchlorosilane for trimethylsilylation. One µL of the derivative extract was then injected for GC/MS analysis using a Trace GC Ultra combined with a TSQ Quantum GC mass spectrometer and a Triplus autosampler (Thermo Electron Corporation, Waltham, MA). A RXI-5-Sil MS w/Integra-Guard^®^ (fused silica) (30 m x 0.25 mm x 0.25 µm) column was used for compound separation. Data acquisition was performed using the software “Xcalibur” (version 4.0) in full scan mode from 50 to 700 m/z. Compounds were identified using their reference standards, retention time, quantifier and qualifier ion, and quantified using their standard calibration curves as mg/kg of fresh leaves.

Lipidic compounds were extracted according to the method of Folch et al. (1957) with some modifications. In the first phase, 0.3 mL of methanol; 0.6 mL of chloroform containing butylated hydroxyl toluene (500 mg/L), and 10 µl of internal standard (stearic acid 100 µg/mL) were used. In a second phase, 0.4 mL of chloroform containing butylated hydroxyl toluene (500 mg/L)/methanol/water 86:14:1 v/v/v was used for the extraction. The combined lower lipid-rich layer of the two extracted phases was finally evaporated to dryness under N_2_ and the samples were re-suspended in 300 µl of acetonitrile/isopropanol/water (65:30:5 v/v/v/) containing cholesterol as the internal standard at a concentration of one µm/mL. Samples were injected into UHPLC Dionex 3000 (Thermo Fischer Scientific Germany) with a RP Ascentis Express column (15 cm x 2.1 mm; 2.7 µm C18), following a 30 min multistep linear gradient as described in Della Corte et al. (2015). The UHPLC system was coupled to an API 5500 triplequadrupole mass spectrometer (Applied Biosystems/MDS Sciex). Compounds were identified based on their reference standard, retention time, and qualifier and quantifier ion, and were quantified (expressed as mg/kg) from linear calibration curves built with standard solutions using Analyst 1.7 software.

Volatile compounds were extracted and injected following the method of Chitarrini et al. (2017a) by using a solid phase micro-extraction. A Trace GC Ultra gas chromatograph coupled to a Quantum XLS mass spectrometer (Thermo Scientific, Electron Corporation, Waltham, MA) was used with a fused silica Stabilwax^®^-DA column (30 m x 0.25 mm i.d. x 0.25 μm) (Restek Corporation, Bellefonte, USA). The headspace was sampled using 2-cm DVB/CAR/PDMS 50/30 μm fiber from Supelco (Bellefonte, PA). Data processing was performed using the software “Xcalibur” (version 4.0). The identification of volatile compounds was done by reference to standards or by comparing retention index and mass spectra using the NIST MS Search 2.3 mass spectral database. Results were semi-quantified as the equivalent of the internal standard (1-heptanol) and expressed as µg/kg of fresh leaves.

Phenolic compounds were extracted according to Vrhovsek et al. (2012) with some modifications made by Chitarrini et al. (2017a). Briefly, the phenolic compounds were extracted from 100 mg of fresh leaves using 0.4 mL of chloroform and 0.6 mL of methanol: water (2:1 v/v); the extraction was repeated by adding 0.6 mL of methanol and water (2:1 v/v) and 0.2 mL of chloroform. The aqueous-methanolic phase of two extractions was collected, combined, and evaporated to dryness under N2. Samples were re-suspended in 500 µl of methanol: water (1:1 v/v) and injected into a Waters Acquity UPLC system (Milford) with a Waters Acquity HSS T3 column (100 mm x 2.1 mm; 1.8 µm). Mass spectrometry detection was performed on a Waters Xevo triple-quadrupole mass spectrometer detector (Milford) with an electrospray ionization (ESI) source (Vrhovsek et al., 2012). Compounds were identified based on their reference standard, retention time, and qualifier and quantifier ion, were quantified using their standard calibration curves and expressed as mg/kg of fresh leaves. Data processing was performed using Waters MassLynx V4.1 software.

### 2.5 Data analysis

A customized R script was used for statistical analysis and data visualization (R Core Team, 2020). To perform multivariate analysis, the metabolomics dataset’s missing values were filled in using median imputation. To account for the anticipated year-to-year fluctuation in the overall metabolic response, the average effect of each year was subtracted for each metabolite/genotype. The base 10 logarithm was used to transform the metabolite concentrations in order to compensate for the heteroscedasticity of the data (van den Berg et al., 2006). Thereafter, metabolic principal component analysis (PCA) was carried out on the resulting multidimensional dataset after UV scaling.

The differential response of the individual metabolites at 24 and 48 hpi was characterized by applying a series of univariate non-parametric tests to the data corrected for the effect of the year. To focus on widely present metabolites, only the compounds detected in eight samples were considered for the univariate analysis. Cohen’s d-effect size was calculated to identify the metabolites that were strongly altered following infection. Statistical significance and effect size were combined in a set of “volcano plots”. Uncorrected *p* < 0.05 and a d > 1 were used as arbitrary thresholds to identify strongly responding metabolites. The “d” values can range from a very small effect (d = 0.01) to a huge one (d = 2.0), as per the study of Sawilowsky (2009). **Supplementary Table 6** displays the “d” values of the identified up-accumulated metabolites, as well as their related effect size and *p* values. No statistical analysis was conducted on the qualitative assessments of leaf health status.

## 3 Results

The results of *E. necator’s* inoculation were phenotypically observed and the best infections (highest sporulation observed on the leaves) were obtained with the modified dry methods of Deliere et al. (2010) and Valdés-Gómez et al. (2011). The dry inoculation method provided more effective infections than the wet inoculation, most likely due to conidia germination being inhibited or reduced in the presence of water, which was reported to have a detrimental influence on the viability and infectivity of powdery mildew conidia (Miclot et al., 2012). Furthermore, high humidity has been demonstrated to have a severe negative influence on grapevine powdery mildew conidia germination (Carroll and Wilcox, 2003). The reduced efficacy of the wet inoculation is most likely due to residual water remaining in the leaves during or after the drying step. Pictures of the inoculated genotypes taken during the OIV-455 score evaluated at 3, 7 and 11 dpi are available as **Supplementary Figures (2- 22**).

Over a two-year period, we were able to identify and quantify/semi-quantify 177 metabolites from four chemical classes. These include 60 primary compounds, 56 volatile organic compounds, 43 phenolic compounds and 17 lipids. In the class of primary compounds, we quantified (26) acids, (13) amino acids, (3) amines, one gamma-butyrolactone, and (17) sugars. Within the lipids, we quantified: (2) glycerophospholipids, one sphingolipid, one glycerolipid, one prenol, and (12) fatty acids. We semi-quantified: (4) acids, (9) alcohols, (8) aldehydes, (6) benzenoids, one ester, (2) other volatile organic compounds, (3) fatty acids, (3) fatty acids esters, one fatty alcohol, one benzofuran, (8) terpenoids, (2) terpenes, (3) ketones, one secondary alcohol, and (4) unknowns for the organic volatile compounds. For phenols, we quantified: (3) benzoic acid derivatives, one coumarin, one dihydrochalcone, (12) flavan-3-ols, one flavanone, (12) flavonols, (3) phenylpropanoids, (8) stilbenes and stilbenoids and two other compounds.

The obtained concentrations of all investigated metabolites for each genotype in both years are presented in **Supplementary Table 2** for primary compounds, in **Supplementary Table 3** for lipids, in **Supplementary Table 4** for VOC(s), and in **Supplementary Table 5** for phenolic compounds.

### 3.1 Resistant and susceptible genotypes reveal different kinetics upon pathogen inoculation

**Figure 3:**
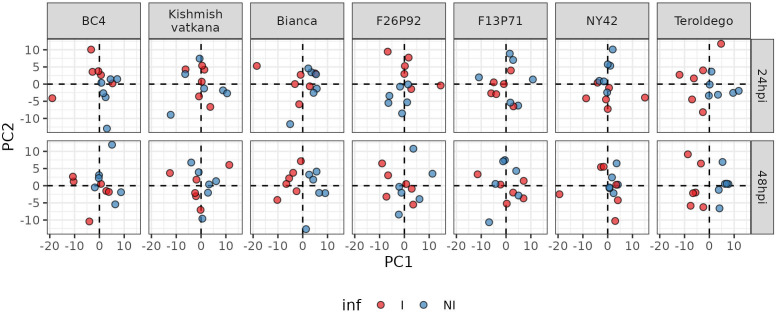
Principal component analysis performed on the log 10-transformed metabolite concentration of 24 and 48hpi samples. Each genotype has three biological replicates (small dots) for each year (2019 and 2021). The red color is for inoculated samples (I, inoculated samples) and the blue is for non-inoculated samples (NI, not inoculated samples).

After the removal of the effect of the year, PCA was used to depict the global metabolite changes of the 177 identified metabolites in response to pathogen inoculation in all seven genotypes for both years (**Figure 3**). The six biological replicates of each genotype (three per year) are represented in the plots as small colored dots (the red color corresponds to the inoculated samples and the blue color to the non-inoculated samples). Samples collected at 24 and 48 dpi were analyzed separately to account for possible differences in response among the different genotypes.

The PCA revealed different timescales for the onset of the metabolic response. In fact, in ‘Bianca’ and ‘Teroldego’, the separation of infected and non-infected samples began at 24 hpi along the first dimension and became very evident at 48 hpi (**Figure 3**). Oddly, ‘Kishmish vatkana’ and F13P71 did not show any separation, neither at 24 hpi nor at 48 hpi (**Figure 3**). BC4, F26P92, and NY42 showed instead a partial separation through the second dimension at 24 hpi, which was no longer observable by 48 hpi (**Figure 3**).

### 3.2 The modulation of classes of compounds upon pathogen inoculation

**Figure 4:**
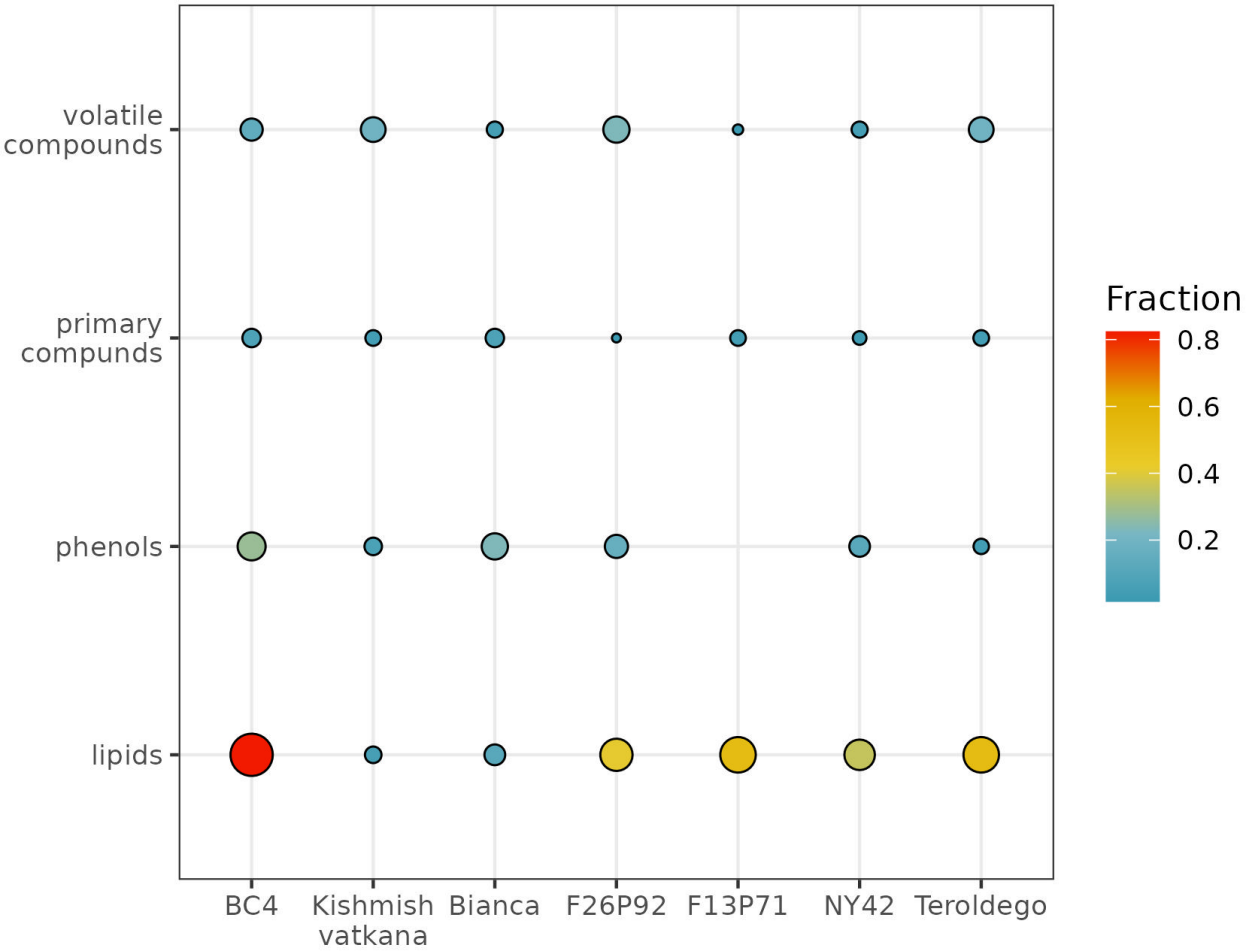
Global visualization of highly modulated metabolites by chemical class (in percentage) in response to *E. necator* inoculation. The size and color intensity of the dots are proportional to the estimated percentage of metabolites modulated in each genotype in both years, based on the total number of identified and quantified/semi-quantified metabolites per class.

To determine to which classes of compounds the metabolites that were responsible for the various sorts of separations between genotypes belong, we analyzed the percentages of compounds per class that had a significant effect after infection (**Figure 4**). The graph represents the percentage of metabolites per each class of compounds that were highly modulated in the plants of each genotype out of the total number of identified and quantified/semi-quantified metabolites per class in both years, as a response to the infection (i.e., 61 primary compounds, 56 volatile organic compounds, 43 phenolic compounds, and 17 lipids).

The class of compounds that were highly modulated due to the infection consisted in lipids. This class showed higher levels compared to the control (non-infected plants), due to the biotic stress in five out of the seven studied genotypes (i.e., BC4, F13P71, F26P92, NY42, and ‘Teroldego’). The estimated percentage of lipids affected in BC4 was around 80%; in F13P71 and in ‘Teroldego’, the percentage of affected lipids decreased to 60% and continued to decrease in F26P92 and NY42 down to 40% reaching 20% in ‘Bianca’ and less than 20% in ‘Kishmish vatkhana’.

Within the class of phenols, the genotype BC4 had the topmost modulated metabolites with a percentage of around 40%. ‘Bianca’, F26P92, and NY42 reached an approximate value of 20%, whereas the modulation of the metabolites in ‘Kishmish vatkana’ and ‘Teroldego’ remained below 20%. An exception was the genotype F13P71, which showed a very low percentage of modulation, not reported in the figure.

The primary compounds exhibited a similar trend of approximately 20% modulated metabolites within the genotypes BC4 and ‘Bianca’, with a slow decrease in F13P71 and ‘Teroldego’. A much lesser percentage was observed in NY42 and F26P92.

The modulation of metabolites in the class of volatile compounds was estimated below 40% for the genotype F26P92, 20% for BC4, ‘Kishmish vatkana’, and ‘Teroldego’; below 20% for NY42, and lower in F13P71.

### 3.3 Modulated metabolites induced by *Erysiphe necator*

**Figure 5:**
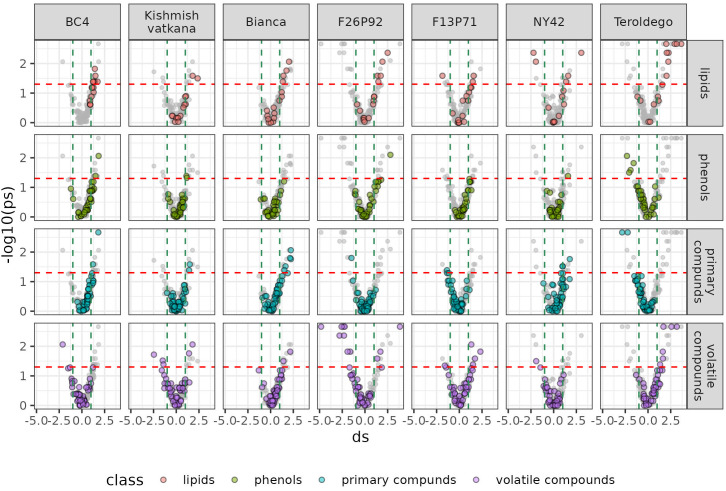
Metabolites significantly modulated by the infection (up- and down- accumulated) by class of compounds in all seven genotypes at 24 hpi in the two years of data analysis (2019–2021). The colors identify the different chemical classes (red for lipids, green for phenols, blue for primary compounds, and violet for volatile organic compounds) and “ds” represents the calculated Cohen’s d values.

**Figure 6:**
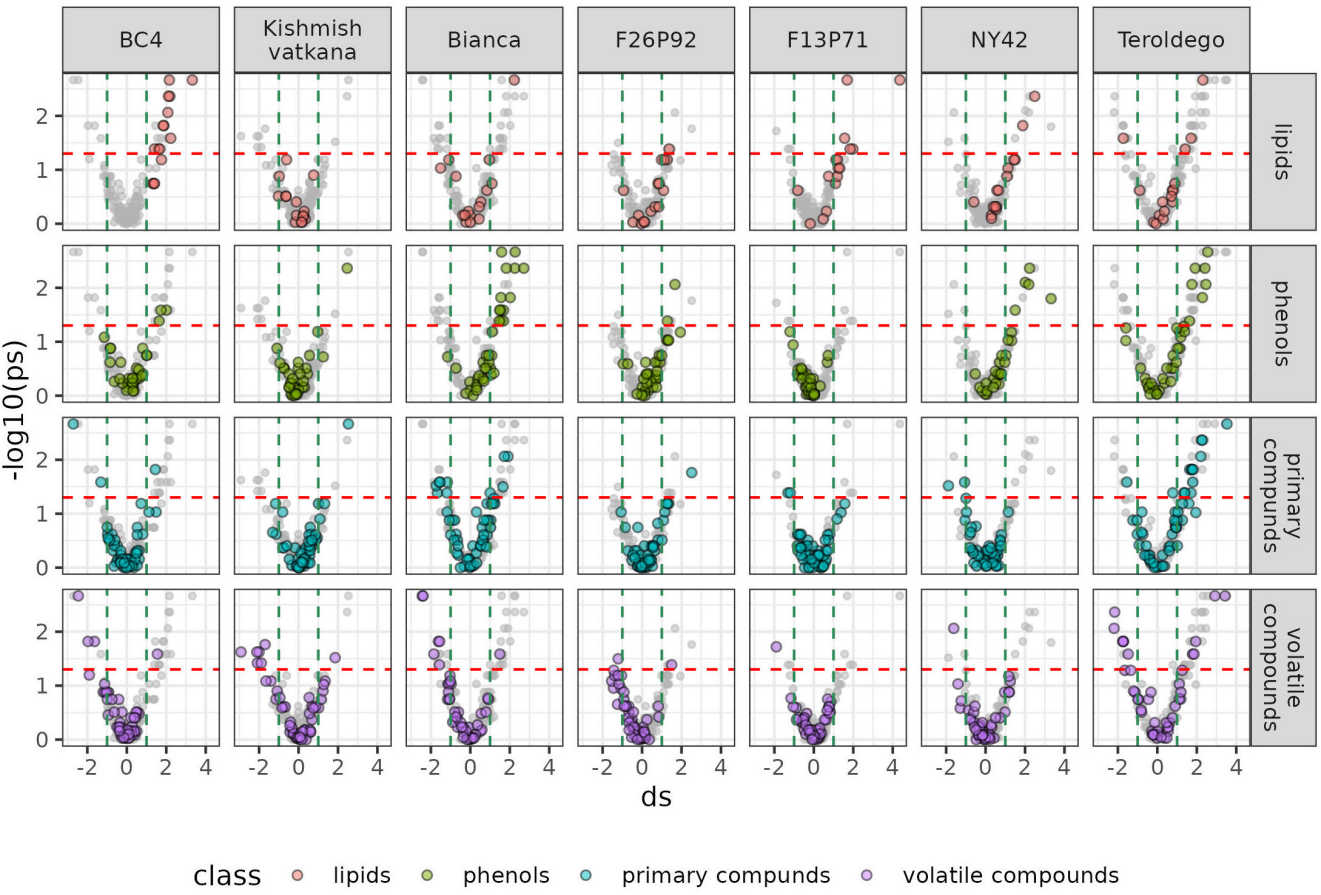
Metabolites significantly modulated by the infection (up- and down- accumulated) by class of compounds in all seven genotypes at 48 hpi in the two years of data analysis (2019–2021). The colors identify the different chemical classes (red for lipids, green for phenols, blue for primary compounds, and violet for volatile organic compounds) and “ds” represents the calculated Cohen’s d values.

We set out to identify specific metabolites that varied during the infection consistently in both years based on the results of the classes of compounds shown above. As discussed in materials and methods, the most relevant metabolites were identified by combining statistical significance (assessed by a univariate test) and strength of the effect (estimated by calculating the effect size). We then presented this information in a series of volcano plots (**Figure 5** and **6**) that highlight the modulation of the distinct classes for each genotype. Positive impact magnitude suggests abundant production (up-accumulation) of the metabolite in infected plants. As a result, a high tail in the volcano’s right arm indicates a favorable metabolic response to infection. The lowered (down-accumulation) quantity of metabolites as a reaction to infection, on the other hand, has a negative effect size. It can be seen graphically as the high tail in the volcano’s left arm.

Overall, **Figure 5** and **6** confirm the trends observed in the initial PCA results, but the plots can be used to get an insight into the classes of metabolites, which are more involved in the response. Generally, it can be noticed that the genotypes ‘Bianca’ and ‘Teroldego’ begin to react at 24 hpi (**Figure 5**) and that the effect becomes much larger at 48 hpi (**Figure 6**), reaching in some cases an effect size value of 2 and even 3 (e.g. phenols in ‘Bianca’ and ‘Teroldego’). In fact, ‘Bianca’ exhibits the onset of an infection response in all four classes of compounds at 24 hpi, which becomes stronger at 48 hpi by producing a large number of up-accumulated phenols, followed by primary compounds and lipids, and several down-accumulation of volatile compounds. ‘Teroldego’ produces primarily up-accumulated chemicals such as lipids and volatiles at 24 hpi, whereas, at 48 hpi, there is a large production of up-accumulated phenols, primary and volatile compounds.

The genotypes F13P71 and ‘Kishmish vatkana’, which appeared not to show major changes in the PCA analysis, showed an up-accumulation in a limited number of lipids and volatiles at 24hpi. At 48hpi, however, ‘Kishmish vatkana’ reestablished an equilibrium that modulated the levels of up-accumulated lipids and volatiles to levels comparable to the non-infected plants of the same genotype. In the case of F13P71, the levels of lipids increased by 48 hpi, while volatiles appeared not to be modulated anymore.

As for genotypes BC4, F26P92 and NY42, they showed the third type of trend in the PCA where a partial separation between infected and non-infected plants was observed at 24 hpi, BC4 up-accumulated lipids and phenolic compounds at 24 hpi and an increase in that up-accumulation at 48 hpi. It also showed an increase in down-accumulation of volatiles from 24 hpi to 48 hpi. F26P92 showed an active reaction in the synthesis of up-accumulated lipids and down-accumulation of volatile compounds only at 24hpi. NY42 showed a rise in lipids and primary compounds at 24hpi only, while phenols highly increased from 24hpi to 48hpi.

**Figure 7:**
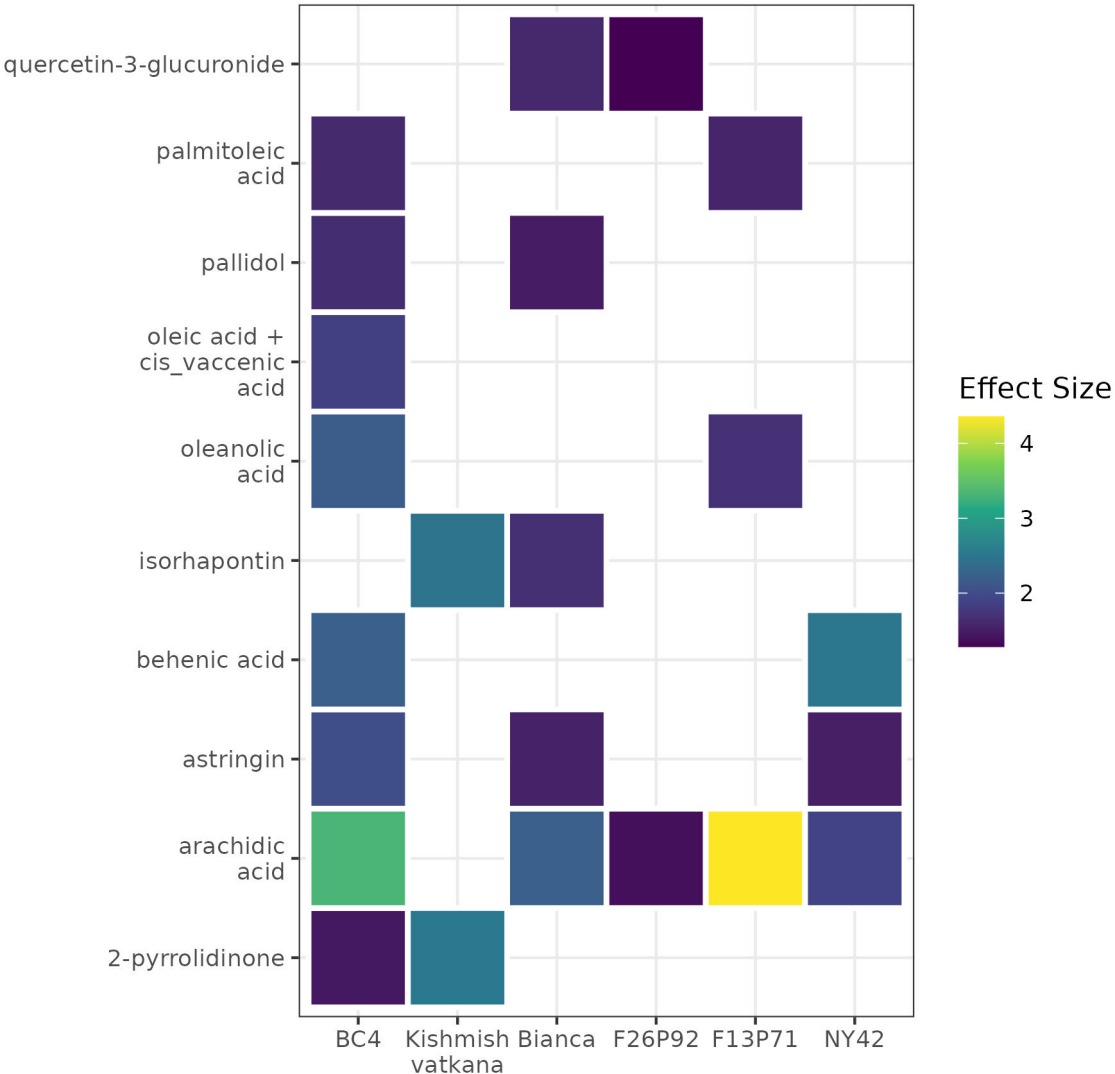
A heat map using color-encoded effect size of the discriminative compounds identified as present in the resistant genotypes and absent in the susceptible genotype at 48 hpi. The colors and their intensities mark the modulation of the metabolites in the resistant genotypes according to the calculated effect size (Cohen’s d test).

A list of modulated metabolites with the highest reaction in terms of effect size and *p-values* is synthesized in **Supplementary Table 6**. Among them, we noticed ten up-accumulated metabolites that might potentially distinguish resistant (partial/total) genotypes from the susceptible genotype at 48hpi, when we know that the pathogen’s infection structures had already interfered with the plant’s metabolome. These metabolites were 2-pyrrolidinone, oleanolic acid, behenic acid, palmitoleic acid, arachidic acid, oleic acid+*cis*_vaccenic acid, pallidol, isorhapontin, quercetin-3-glucoronide, and astringin. Their presence and/or absence in the genotypes is outlined in the **Figure 7**. The changes of the discriminative compounds at 0hpi, 24hpi and 48hpi for all genotypes based on the corrected concentration values as described in materials and methods are displayed in **Supplementary Figure 1**.

## 4 Discussion

In nature, plants protect themselves mostly through mechanical means (spines, trichomes, thick cuticles, and hard or sticky surfaces) and the emission of a variety of poisonous, repellent or unattractive compounds. Plants produce a wide range of metabolites through the latter protection strategy, including fundamental metabolites such as primary compounds and lipids, as well as secondary metabolites like phenolic and volatile organic compounds (Mazid et al., 2011). Secondary metabolism is known to play a defensive role against predators, parasites and diseases (Ali et al., 2010), and primary metabolism, in addition to controlling plant growth, development and reproduction, contributes to plant defense as a source of energy and by signaling molecules that directly or indirectly trigger defense responses (Wolfender et al., 2013).

In this study, we examined the contribution of secondary and primary metabolic components in mediating plant defense responses in resistant grapevine genotypes inoculated by *E. necator*. To the best of our knowledge, this is the first study to look at the responsiveness of multiple classes of metabolites in varieties with one gene of resistance versus varieties with multiple barriers of resistance against powdery mildew.

Our findings indicated that diverse grapevine genotypes react with different time scales to infection. Interestingly, metabolic response (primary and secondary) was more active in the partial and total resistant varieties (i.e., ‘Bianca’, F26P92, NY42, and BC4) and in the susceptible cultivar ‘Teroldego’ compared to the partially resistant mono-locus (‘Kishmish vatkhana’) and the totally resistant pyramided variety (F13P71) (**Table 1**). ‘Bianca’, as well as ‘Teroldego’, showed metabolic variability caused by pathogen inoculation at both time points, while F26P92 and NY42 showed metabolic variability only at 24 hpi. This could be explained by the studies of Feechan et al. (2015) and Pap et al. (2016), which indicated that the existence of several resistance genes or loci does not result in a stronger resistance response for all genotypes, thereby suggesting that combinations of loci such as *Ren3Ren9* do not always have additive effects (Zendler et al., 2020) when compared to the *Run1Ren1* combination that produces an additive effect (Agurto et al., 2017). The method of activating a gene is complicated since just having the gene is not enough; instead, transcription factors are required (Agurto et al., 2017).

Such responses have been observed in some genotypes carrying the combination of *Ren3* and *Ren9*, which did not generate an immune response that has an advantage in terms of the intensity or speed of the response compared to *Ren3* alone (Zini et al., 2019; Zendler et al., 2020). The presence of these loci (*Ren3* and *Ren9*) in all three of the pyramided genotypes, ‘Bianca’, F26P92, and NY42 (**Table 1**), could explain our PCA results, which revealed that ‘Bianca’ had a metabolic variability caused by pathogen inoculation at both time points similar to ‘Teroldego’, followed by F26P92 and NY42, which showed metabolic variability only at 24 hpi (**Figure 3**).

On the other hand, studies showed that combinations of *Run1Ren1* and *Run1Ren2* have an additive effect as the combination of both genes/loci generated a stronger immune response than the one triggered by each one individually, however, this effect has been proven to be genotype dependent (Agurto et al., 2017). In fact, in our study, the genotypes F13P71 and NY42 showed little to partial metabolic variability despite possessing the loci *Run1Ren1* and *Run1Ren2*, respectively. Moreover, other studies showed that by powdery mildew isolates could overcome, in some cases, *Run1* resistance (Cadle-Davidson et al., 2011; Schneider et al., 2019). This could explain the observed metabolic variability in F13P71. Furthermore, the additive effect of *Run1Ren2* can be race-specific (Feechan et al., 2015) and in addition, the existence of the other two extra loci in the genotype NY42, might interfere with the metabolomics response to the pathogen. All these factors contribute to the complexity of the effects of resistance genes in the metabolic variability of infected grapevine genotypes, requiring additional research.

Considering all these aspects, it seems that the level of resistance (partial or total) of the loci is more important than their numbers. The level of resistance is referred to as “total” when there are greatly suppressed symptoms or no observable symptoms of infection at all and “partial” when there is a decrease in symptoms but not a complete disappearance (Julius Kühn-Institut, 2022; Sosa-Zuniga et al., 2022). This is corroborated in our study by the assessment of the OIV-455 descriptor at 7 days after the artificial infection (**Supplementary Table 1**).

We found for ‘Kishmish vatkana’, a genotype with partial resistance, a high level of resistance (OIV-455 = 7) and for F13P71, a genotype with total resistance, a very high level of resistance (OIV-455 = 9). Indeed, an 84% decrease in the number of cells the fungus invaded has been observed among the responses brought on by *Ren1* (‘Kishmish vatkana’). Other reactions include the induction of PCD (programmed cell death), the development of callose deposits at 48 hpi, and the promotion of ROS (reactive oxygen species) at 96 hpi (Agurto et al., 2017). A more intense defense response was likewise observed in genotypes carrying *Run1Ren1*, such as F13P71, in terms of ROS production, callose accumulation and PCD (Agurto et al., 2017).

NY42 and F26P92, genotypes with partial resistance, scored a high level of resistance (OIV-455 = 7) and BC4, a genotype with total resistance, was assessed as having a very high level of resistance (OIV-455 = 9). Possamai et al. (2021) observed in genotypes carrying *Run1Ren2* loci such as NY42 a significant decrease in colony formation, and Feechan et al. (2015) showed that *Ren2* confers partial resistance on plants by inducing an efficient immune response that prevents fungal sporulation. Rapid programmed cell death, which hinders the growth of secondary hyphae and sporulation, is one of the immunological responses inflicted by *Run1* (BC4) on resistant plants. A quick HR is seen at 48 hpi in cells where the fungus developed secondary hyphae as evidenced by the rise in ROS and the appearance of PCD. The buildup of callose deposits at the *E. necator* infection site is another reaction caused by *Run1* (Agurto et al., 2017). *Ren3Ren9* (F26P92) elicits similarly high resistance responses (Zendler et al., 2020), with a high level of resistance score (OIV-455 = 8) assigned to ‘Bianca’, a partial resistant genotype carrying the exact same loci (*Ren3Ren9)*. As expected, the susceptible genotype ‘Teroldego’ was assessed as having a very low level of resistance (OIV-455 = 1). As far as primary metabolites are concerned, powdery mildew induced changes mainly in the class of lipids (**Figure 4**). Lipids are recognized to be important components of plant cell membranes that provide energy for metabolic activities. In recent years, there has been increasing evidence that lipids play a role in combating biotic stress, such as powdery mildew. Lim et al. (2017) showed that lipids regulate the PCD response during pathogen defense, as well as membrane fluidity, stability, and permeability during plant responses to microbial pathogens. The accumulation of C16:0 might be used to produce C18 fatty acids. Also higher DBI may account for an increase in chloroplasts’ membrane fluidity that may be crucial to avoid any damage in the photosynthetic machinery with inevitable effects on the energy transduction pathways and primary productivity (Laureano et al., 2018; Laureano et al., 2021). Moreover, lipids play important signaling roles also in plant defense and ROS regulating levels. Because of the various functions of lipids, Della Corte et al. (2015) observed that their abundance in plants is influenced by genotype and phenotype. Thus, the fluctuating lipid levels observed in the various resistant genotypes tested may be attributed in part to this aspect as a result of *E.necator* inoculation.

The role of primary metabolic pathways in the regulation of plant defense responses is not very well known. Mainly, all primary compounds function as signaling molecules that trigger defense responses through signal transduction and pathogen recognition processes (Madiha et al., 2019). The accumulation of the primary compounds in our study, which was comparable to the susceptible genotype, made us lend support to the idea that susceptible plants initiate a basal defense similar to the response in resistant plants, but insufficient in timing and/or intensity to limit disease progression, as observed by Marsh et al. (2010). Similarly, there is a clear alteration of primary compounds in the defense against powdery mildew in resistant genotypes, but the amount raises the question of whether this modulation is a result of resistance or a normal plant reaction.

One of the most important functions of phenolic compounds as secondary metabolites is an antibacterial activity in plants, which acts as a barrier against pathogens like *E. necator*. Their accumulation in plants is associated with host resistance (Atak, 2017). However, it is noteworthy that Keller (2015) found that despite some *Viti*s species (such as *V.cinerea* and *V.champinii*) exhibiting pathogen resistance, the buildup of stilbenes, the most well-known class of phenolic defense chemicals, did not occur in these plants. This finding could support the hypothesis that metabolite accumulation is not totally linked to the number of loci present in the resistance genotypes. Such was the case in our study where the pyramided genotype F13P71 accumulated very low levels of phenolic compounds. The same genotype displayed low levels of volatile organic compounds (VOCs). Thus, similar assumptions could be made also about VOCs, but further research is needed to confirm it.

Although some chemical classes in some of our resistant varieties showed similar reactions to the susceptible genotype, it should be noted that the resistant genotypes nonetheless produce a number of up-accumulated metabolites that were not found in the susceptible ‘Teroldego’, with the exception of a few whose calculated effect sizes were smaller than in the resistant genotypes. The study of Viret et al. (2018) showed that the induction and accumulation of defensive metabolites increase only during the pathogen’s infectious structure development, which takes around 24 hours (Boddy, 2016). This was noticed in the pyramided genotypes in which the metabolite overaccumulation had a significantly larger impact size at 24 hpi than at 48hpi, when their modulation appeared to subside, except for ‘Bianca’. In our earlier research, we provided evidence that *P. viticola* caused an early modulation in pyramided genotypes, which began earlier, between 0 and 12 hpi, and peaked at 48hpi. Even though the current work studies *E. necator* and genotypes that carry different loci than the prior study, we can presume that a similar but somewhat different reaction happened for this study as well. On the other hand, the up-accumulation of metabolites in mono-locus genotypes was shown to be established at 24 hpi and to become stronger at 48hpi. The same finding was obtained in the work of Chitarrini et al. (2017a), in which the plant defense systems were activated 48 hours after inoculation.

Investigating the biological relevance of the ten compounds found as discriminative between resistant and susceptible genotype (**Figure 7**), we found out that pallidol, oleic acid+*cis* vaccenic acid and astringin were already discussed as potential biomarkers of resistance in our previous study (Ciubotaru et al., 2021) due to their role in activating plant defense response. Moreover, pallidol has been in some cases linked to the grapevine’s response to fungal attack (Pezet et al., 2004; Jean-Denis et al., 2006). We have also found that the remaining seven- up-accumulated metabolites contribute to plant defense. Isorhapontin, like pallidol and astringin, belongs to the class of stilbenes and stilbenoids, and it has been demonstrated that this class accumulates in larger concentrations in disease-resistant cultivars than in susceptible cultivars, due to its role in plants that inhibits fungal growth (Chitarrini et al., 2017b; Vezzulli et al., 2019). Similarly, the increased accumulation of fatty acids in the plant metabolome, specifically behenic acid, palmitoleic acid, arachidic acid and oleic acid+*cis* vaccenic suggests that these fatty acids are involved in intracellular signaling processes as well as desaturases-mediated membrane fluidity adjustment (He and Ding, 2020; Ciubotaru et al., 2021). The fatty acid desaturase 7 (FAD7) and fatty acid desaturase 8 (FAD8) genes, which play a key role in the synthesis of fatty acids, have also been linked to protective mechanisms (Rojas et al., 2014; Cavaco et al., 2021). Last but not least, oleanolic acid is known to play a role in plants’ defense mechanisms against pathogens and water loss (Gudoityte et al., 2021), whereas quercetin is a powerful antioxidant that effectively protects plants from a variety of biotic and abiotic challenges (Singh et al., 2021). Our findings confirm previous research about the importance of these compounds in disease resistance because of their different roles in plant defense.

## 5 Conclusions

Many metabolomics studies have been conducted on understanding the mechanism of grapevine defense, mainly on downy mildew, but few on powdery mildew. Thus, we designed this study as a promising endeavor in order to contribute to a better understanding of plant defense mechanisms. To our knowledge, this is the first time that metabolic investigations of the most important classes of compounds with a role in plant defense were carried out in artificially inoculated genotypes with mono-locus and pyramided resistance in order to characterize the host’s response to the infection of *E. necator*.

Overall, the results of this study indicate that how cultivars behaved to pathogen attack can be linked to genotype and/or resistant loci differences; however, resistance is not exclusively related to *Run/Ren* loci. Additionally, although it cannot be strictly classified as a connection, we saw similar metabolomic responses in our experiment between the mono-locus and pyramided genotypes that share the exact *Run/Ren* loci. Therefore, additional transcriptome studies are required to fully comprehend the unfavorable interaction between these resistant loci and *E. necator*. Further research is needed also to validate the molecules identified as biologically relevant compounds produced during the pathogen-host interaction and recommended as possible biomarkers for resistance to *E. necator*. In terms of plant resistance strength against powdery mildew, our findings show no direct relationship between the number of resistance loci present in plants and the production of metabolites recommended as resistance biomarkers.

The findings of this study add to our understanding of plant defense mechanisms and call for more metabolomics research, as well as additional complementary omics research to clarify which genes are responsible for powdery mildew resistance and how they function in the majority of *Run* and *Ren* loci, as only one study in this area has been conducted. The integration of transcriptomics and metabolomics data can be exploited to uncover commonalities and differences between diverse R-gene-mediated resistances to *E. necator.* This approach will enable breeders to choose more reliable genotypes for marker-assisted breeding by using genetic and biochemical markers.

## Data availability statement

The original contributions presented in the study are included in the article/**Supplementary Material**. Further inquiries can be directed to the corresponding author.

## Author contributions

RMC, GC, LZ, MS, and UV designed the experiment. MS and SV provided the plant material. RMC, SV and LZ performed the experiment. RMC did the extractions and analytical analysis. PF, RMC, and UV conducted the data treatment, statistical analysis and data visualization. RMC wrote the original draft preparation of the manuscript. UV, SV, PF, MO, PR and GC did the review and editing. UV, MO, and PR supervised the project. All authors discussed the results and implications and commented on the manuscript at all stages. All authors contributed to the article and approved the submitted version.

## References

[B1] Agurto M. Schlechter R. O. Armijo G. Solano E. Serrano C. Contreras. (2017). *RUN1* and *REN1* pyramiding in grapevine (*Vitis vinifera* cv. crimson seedless) displays an improved defense response leading to enhanced resistance to powdery mildew (*Erysiphe necator*). Front. Plant Sci. 8. doi: 10.3389/fpls.2017.00758 PMC5427124 28553300

[B2] Ali K. Maltese F. Choi Y. H. Verpoorte R. (2010). Metabolic constituents of grapevine and grape-derived products. Phytochem. Rev. 9, 357–378. doi: 10.1007/s11101-009-9158-0 20835385 PMC2928446

[B3] Atak A. (2017). Determination of downy mildew and powdery mildew resistance of some grape cultivars. South Afr. J. Enology Viticulture 38 (1), 11–17. doi: 10.21548/38-1-671

[B4] Atak A. (2022). “New perspectives in grapevine breeding,” in Plant breeding - new perspectives. Ed. Wang H. (Rijeka, Croatia: IntechOpen). doi: 10.5772/intechopen.105194

[B5] Atak A. Göksel Z. Yılmaz Y. (2021). Changes in major phenolic compounds of seeds, skins, and pulps from various vitis spp. and the effect of powdery and downy mildew diseases on their levels in grape leaves. Plants 10, 2554. doi: 10.3390/plants10122554 34961024 PMC8703439

[B6] Atak A. Şen A. (2021). A grape breeding programme using different vitis species. Plant Breed. 140 (6), 1136–1149. doi: 10.1111/pbr.12970

[B7] Boddy L. (2016). “Pathogens of autotrophs,” in The fungi (Oxford, UK: Academic Press), 245–292.

[B8] Cadle-Davidson L. Mahanil S. Gadoury D. M. Kozma P. Reisch B. I. (2011). Natural infection of *Run1*-positive vines by naïve genotypes of *Erysiphe necator* . Vitis 50 85, 173–175.

[B9] Carroll J. E. Wilcox W. F. (2003). Effects of humidity on the development of grapevine powdery mildew. Phytopathology 93, 1137–1144. doi: 10.1094/PHYTO.2003.93.9.1137 18944098

[B10] Cavaco A. R. Laureano G. Cunha J. Eiras-Dias J. Matos A. R. Figueiredo A. (2021). Fatty acid modulation and desaturase gene expression are differentially triggered in grapevine incompatible interaction with biotrophs and necrotrophs. Plant Physiol. Biochem. 163, 230–238. doi: 10.1016/j.plaphy.2021.04.001 33862502

[B11] Chitarrini G. Soini E. Riccadonna S. Franceschi P. Zulini L. Masuero D.. (2017a). Identification of biomarkers for defense response to *Plasmopara viticola* in a resistant grape variety. Front. Plant Sci. 8. doi: 10.3389/fpls.2017.01524 PMC5591819 28928759

[B12] Chitarrini G. Zulini L. Masuero D. Vrhovsek U. (2017b). Lipid, phenol and carotenoid changes in 'Bianca' grapevine leaves after mechanical wounding: a case study. Protoplasma 254 (6), 2095–2106. doi: 10.1007/s00709-017-1100-5 28324165

[B13] Ciubotaru R. M. Franceschi P. Zulini L. Stefanini M. Škrab D. Rossarolla M. D.. (2021). Mono-locus and pyramided resistant grapevine cultivars reveal early putative biomarkers upon artificial inoculation with *Plasmopara viticola.* front. Plant Sci. 12. doi: 10.3389/fpls.2021.693887 PMC8281963 34276743

[B14] Deliere L. Miclot A. S. Sauris P. Rey P. Calonnec A. (2010). Efficacy of fungicides with various modes of action in controlling the early stages of an *Erysiphe necator*-induced epidemic. Pest Manag Sci. 66 (12), 1367–1373. doi: 10.1002/ps.2029 20949548

[B15] Della Corte A. Chitarrini G. Di Gangi I. M. Masuero D. Soini E. Mattivi F.. (2015). A rapid LC-MS/MS method for quantitative profiling of fatty acids, sterols, glycerolipids, glycerophospholipids and sphingolipids in grapes. Talanta 140, 52–61. doi: 10.1016/j.talanta.2015.03.003 26048823

[B16] Dry I. B. Thomas M. R. (2015). Fast-tracking grape breeding for disease resistance. Wine Vitic. J. 5, 52–55.

[B17] Feechan A. Anderson C. Torregrosa L. Jermakow A. Mestre P. Wiedemann-Merdinoglu S.. (2013). Genetic dissection of a TIR-NB-LRR locus from the wild north American grapevine species *Muscadinia rotundifolia* identifies paralogous genes conferring resistance to major fungal and oomycete pathogens in cultivated grapevine. Plant J. 76, 661–674. doi: 10.1111/tpj.12327 24033846

[B18] Feechan A. Kabbara S. Dry I. B. (2011). Mechanisms of powdery mildew resistance in the *Vitaceae* family. Mol. Plant Pathol. 12, 263–274. doi: 10.1111/j.1364-3703.2010.00668.x 21355998 PMC6640449

[B19] Feechan A. Kocsis M. Riaz S. Zhang W. Gadoury D. M. Walker M. A.. (2015). Strategies for *RUN1* deployment using *RUN2* and *REN2* to manage grapevine powdery mildew informed by studies of race specificity. Phytopathology 105, 1104–1113. doi: 10.1094/PHYTO-09-14-0244-R 26039639

[B20] Folch J. Lees M. Sloane Stanley G. H. (1957). A simple method for the isolation and purification of total lipids from animal tissues. J. Biol. Chem. 226, 497–509. doi: 10.1016/S0021-9258(18)64849-5 13428781

[B21] Gadoury D. M. Cadle-Davidson L. Wilcox W. F. Dry I. B. Seem R. C. Milgroom M. G. (2012). Grapevine powdery mildew (*Erysiphe necator*): A fascinating system for the study of the biology, ecology and epidemiology of an obligate biotroph: Grapevine powdery mildew. Mol. Plant Pathol. 13, 1–16. doi: 10.1111/j.1364-3703.2011.00728.x 21726395 PMC6638670

[B22] Gudoityte E. Arandarcikaite O. Mazeikiene I. Bendokas V. Liobikas J. (2021). Ursolic and oleanolic acids: Plant metabolites with neuroprotective potential. Int. J. Mol. Sci. 22, 4599. doi: 10.3390/ijms22094599 33925641 PMC8124962

[B23] Gur L. Cohen Y. Frenkel O. Schweitzer R. Shlisel M. Reuveni M. (2022). Mixtures of macro and micronutrients control grape powdery mildew and alter berry metabolites. Plants 11, 978. doi: 10.3390/plants11070978 35406958 PMC9002579

[B24] He M. Ding N. Z. (2020). Plant unsaturated fatty acids: Multiple roles in stress response. Front. Plant Sci. 11. doi: 10.3389/fpls.2020.562785 PMC7500430 33013981

[B25] Hoffmann S. Di Gaspero G. Kovács L. Howard S. Kiss E. Galbács Z.. (2008). Resistance to *Erysiphe necator* in the grapevine ‘Kishmish vatkana’ is controlled by a single locus through restriction of hyphal growth. Theor. Appl. Genet. 116, 427–438. doi: 10.1007/s00122-007-0680-4 18064436

[B26] Jean-Denis J. B. Pezet R. Tabacchi R. (2006). Rapid analysis of stilbenes and derivatives from downy mildew-infected grapevine leaves by liquid chromatography-atmospheric pressure photoionisation mass spectrometry. J. Chromatogr. 1112 (1-2), 263–268. doi: 10.1016/j.chroma.2006.01.060 16458906

[B27] Julius Kühn-Institut (2022). Federal research centre for cultivated plants (JKI), institute for grapevine breeding (Geilweilerhof (ZR). Available at: www.vivc.de/loci (Accessed October 22nd, 2022).

[B28] Keller M. (2015). The science of grapevines: Anatomy and physiology (London, UK: Academic Press).

[B29] Laureano G. Cavaco A. R. Matos A. R. Figueiredo A. (2021). Fatty acid desaturases: Uncovering their involvement in grapevine defence against downy mildew. Int. J. Mol. Sci. 22 (11), 5473. doi: 10.3390/ijms22115473 34067363 PMC8196838

[B30] Laureano G. Figueiredo J. Cavaco A. R. Duarte B. Caçador I. Malhó R.. (2018). The interplay between membrane lipids and phospholipase a family members in grapevine resistance against plasmopara viticola. Sci. Rep. 8 (1), 14538. doi: 10.1038/s41598-018-32559-z 30266912 PMC6162203

[B31] Lim G. H. Singhal R. Kachroo A. Kachroo P. (2017). Fatty acid-and lipid-mediated signaling in plant defense. Annu. Rev. Phytopathol. 4; 55, 505–536. doi: 10.1146/annurev-phyto-080516-035406 28777926

[B32] Madiha Z. Mahpara F. Yasir S. Muhammad H. Zafar H. A. Khalid A. K. (2019). Role of primary metabolites in plant defense against pathogens. Microbial Pathogenesis 137:103728. doi: 10.1016/j.micpath.2019.103728 31499183

[B33] Maia M. Ferreira A. E. N. Nascimento R. Monteiro F. Traquete F. Marques A. P.. (2020). Integrating metabolomics and targeted gene expression to uncover potential biomarkers of fungal/oomycetes-associated disease susceptibility in grapevine. Sci. Rep. 10, 15688. doi: 10.1038/s41598-020-72781-2 32973337 PMC7515887

[B34] Marsh E. Alvarez S. Hicks L. M. Barbazuk W. B. Qiu W. Kovacs L.. (2010). Changes in protein abundance during powdery mildew infection of leaf tissues of Cabernet sauvignon grapevine (*Vitis vinifera* l.). Proteomics 10, 2057–2064. doi: 10.1002/pmic.200900712 20232356

[B35] Mazid M. A. Khan T. A. Mohammad F. (2011). Role of secondary metabolites in defense mechanisms of plants. Biol. Med. 3, 232–249.

[B36] McDonald B. A. Linde C. (2002). Pathogen population genetics, evolutionary potential, and durable resistance. Annu. Rev. Phytopathol. 40, 349–379. doi: 10.1146/annurev.phyto.40.120501.101443 12147764

[B37] Merdinoglu D. Schneider C. Prado E. Wiedemann-Merdinoglu S. Mestre P. (2018). Breeding for durable resistance to downy and powdery mildew in grapevine. Oeno One 52, 189–195. doi: 10.20870/oeno-one.2018.52.3.2116

[B38] Miclot A. S. Wiedemann-Merdinoglu S. Duchêne E. Merdinoglu D. Mestre P. (2012). A standardized method for the quantitative analysis of resistance to grapevine powdery mildew. Eur. J. Plant Pathol. 133, 483–495. doi: 10.1007/s10658-011-9922-z

[B39] Mundt C. C. (2018). Pyramiding for resistance durability: Theory and practice. Phytopathology 108, 792–802. doi: 10.1094/PHYTO-12-17-0426-RVW 29648947

[B40] Pap D. Riaz S. Dry I. B. Jermakow A. Tenscher A. C. Cantu D.. (2016). Identification of two novel powdery mildew resistance loci, *Ren6* and *Ren7*, from the wild Chinese grape species *Vitis piasezkii* . BMC Plant Biol. 16, 170. doi: 10.1186/s12870-016-0855-8 27473850 PMC4966781

[B41] Peressotti E. Wiedemann-Merdinoglu S. Delmotte F. Bellin D. Di Gaspero G. Testolin R.. (2010). Breakdown of resistance to grapevine downy mildew upon limited deployment of a resistant variety. BMC Plant Biol. 10, 147. doi: 10.1186/1471-2229-10-147 20633270 PMC3095292

[B42] Pertot I. Gessler C. (2006). “Potential use and major constrains in grapevine powdery and downy mildew biocontrol. efficacy of KBV 99-01 against *Erysiphe necator* and *Plasmopara viticola* ,” in Proceedings of the 5th international workshop on grapevine downy and powdery mildew(San Michele all’Adige, Italy: SafeCrop Centre Istituto Agrario di San Michele all'Adige), 18–23.

[B43] Pezet R. Gindro K. Viret O. Richter H. (2004). Effects of resveratrol, viniferins and pterostilbene on *Plasmopara viticola* zoospore mobility and disease development. Vitis 43, 145–148. doi: 10.5073/vitis.2004.43.145-148

[B44] Pimentel D. Amaro R. Erban A. Mauri N. Soares F. Rego C.. (2021). Transcriptional, hormonal, and metabolic changes in susceptible grape berries under powdery mildew infection. J. Exp. Bot. 72, 6544–6569. doi: 10.1093/jxb/erab258 34106234

[B45] Pirrello C. Mizzotti C. Tomazetti T. C. Colombo M. Bettinelli P. Prodorutti D.. (2019). Emergent ascomycetes in viticulture: An interdisciplinary overview. Front. Plant Sci. 22;10. doi: 10.3389/fpls.2019.01394 PMC6883492 31824521

[B46] Possamai T. Wiedemann-Merdinoglu S. Merdinoglu D. Migliaro D. De Mori G. Cipriani G.. (2021). Construction of a high-density genetic map and detection of a major QTL of resistance to powdery mildew (*Erysiphe necator* sch.) in Caucasian grapes (*Vitis vinifera* l.). BMC Plant Biol. 21, 528. doi: 10.1186/s12870-021-03174-4 34763660 PMC8582213

[B47] Qiu W. Feechan A. Dry I. (2015). Current understanding of grapevine defense mechanisms against the biotrophic fungus (*Erysiphe necator*), the causal agent of powdery mildew disease. Hortic. Res. 2, 15020. doi: 10.1038/hortres.2015.20 26504571 PMC4595975

[B48] R Core Team (2020). A language and environment for statistical computing (Vienna: R Foundation for Statistical Computing). Available at: https://www.R-project.org/.

[B49] Rienth M. Vigneron N. Walker R. P. Castellarin S. D. Sweetman C. Burbidge C. A.. (2021). Modifications of grapevine berry composition induced by main viral and fungal pathogens in a climate change scenario. Front. Plant Sci. 12. doi: 10.3389/fpls.2021.717223 PMC8693719 34956249

[B50] Rojas C. M. Senthil-Kumar M. Tzin V. Mysore K. S. (2014). Regulation of primary plant metabolism during plant-pathogen interactions and its contribution to plant defense. Front. Plant Sci. 10;5. doi: 10.3389/fpls.2014.00017 PMC3919437 24575102

[B51] Sawilowsky S. S. (2009). New effect size rules of thumb. J. Modern Appl. Stat. Methods 8:26, 597–599. doi: 10.22237/jmasm/1257035100

[B52] Schneider C. Onimus C. Prado E. Dumas V. Wiedemann-Merdinoglu S. Dorne M. A.. (2019). INRA-ResDur: the French grapevine-breeding programme for durable resistance to downy and powdery mildew. Acta Hortic. 1248, 207–214. doi: 10.17660/ActaHortic.2019.1248.30

[B53] Singh P. Arif Y. Bajguz A. Hayat S. (2021). The role of quercetin in plants. Plant Physiol. Biochem. 166, 10–19. doi: 10.1016/j.plaphy.2021.05.023 34087741

[B54] Sosa-Zuniga V. Vidal Valenzuela A. Barba P. Espinoza Cancino C. Romero-Romero J. L. Arce-Johnson P. (2022). Powdery mildew resistance genes in vines: An opportunity to achieve a more sustainable viticulture. Pathogens 11, 703. doi: 10.3390/pathogens11060703 35745557 PMC9230758

[B55] Valdés-Gómez H. Gary C. Cartolaro P. Lolas-Caneo M. Calonnec A. (2011). Powdery mildew development is positively influenced by grapevine vegetative growth induced by different soil management strategies. Crop Prot. 30- 9, 1168–1177. doi: 10.1016/j.cropro.2011.05.014

[B56] van den Berg R. A. Hoefsloot H. C. Westerhuis J. A. Smilde A. ,. K. van der Werf M. J. (2006). Centering, scaling, and transformations: improving the biological information content of metabolomics data. BMC Genomics 7, 142. doi: 10.1186/1471-2164-7-142 16762068 PMC1534033

[B57] Vezzulli S. Malacarne G. Masuero D. Vecchione A. Dolzani C. Goremykin V.. (2019). The Rpv3-3 haplotype and stilbenoid induction mediate downy mildew resistance in a grapevine interspecific population. Front. Plant Sci. 10. doi: 10.3389/fpls.2019.00234 PMC6414455 30894868

[B58] Viret O. Spring J. L. Gindro K. (2018). Stilbenes: biomarkers of grapevine resistance to fungal diseases. Oeno One 52, 235–240. doi: 10.1186/1471-2164-7-142

[B59] Volynkin V. Vasylyk I. Volodin V. Grigoreva E. Karzhaev D. Lushchay E.. (2021). The assessment of agrobiological and disease resistance traits of grapevine hybrid populations (*Vitis vinifera* l. × *Muscadinia rotundifolia* michx.) in the climatic conditions of Crimea. Plants 10 (6), 1215. doi: 10.3390/plants10061215 34203712 PMC8232157

[B60] Vrhovsek U. Masuero D. Gasperotti M. Franceschi P. Caputi L. Viola R.. (2012). A versatile targeted metabolomics method for the rapid quantification of multiple classes of phenolics in fruits and beverages. J. Agric. Food Chem. 60, 8831–8840. doi: 10.1021/jf2051569 22468648

[B61] Welter L. J. Göktürk-Baydar N. Akkurt M. Maul E. Eibach R. Töpfer R.. (2007). Genetic mapping and localization of quantitative trait loci affecting fungal disease resistance and leaf morphology in grapevine (*Vitis vinifera* l). Mol. Breed. 20, 359–374. doi: 10.1007/s11032-007-9097-7

[B62] Welter L. J. Tisch C. Kortekamp A. Topper R. Zyprian E. (2017). Powdery mildew responsive genes of resistant grapevine cultivar 'Regent'. Vitis 56, 181–188. doi: 10.5073/vitis.2017.56.181-188

[B63] Wolfender J. L. Rudaz S. Choi Y. H. Kim H. K. (2013). Plant metabolomics: from holistic data to relevant biomarkers. Curr. Med. Chem. 20 (8), 1056–1090. doi: 10.2174/0929867311320080009 23210790

[B64] Yin W. Wang X. Liu H. Wang Y. van Nocker S. Tu M.. (2022). Overexpression of *VqWRKY31* enhances powdery mildew resistance in grapevine by promoting salicylic acid signaling and specific metabolite synthesis. Horticulture Res. 9, 64. doi: 10.1093/hr/uhab064 PMC8944305 35043152

[B65] Zendler D. Schneider P. Töpfer R. Zyprian E. (2017). Fine mapping of *Ren3* reveals two loci mediating hypersensitive response against *Erysiphe necator* in grapevine. Euphytica 213, 68. doi: 10.1007/s10681-017-1857-9

[B66] Zendler D. Töpfer R. Zyprian E. (2020). Confirmation and fine mapping of the resistance locus *Ren9* from the grapevine cultivar ‘Regent’. Plants 10, 24. doi: 10.3390/plants10010024 33374373 PMC7823669

[B67] Zini E. Dolzani C. Stefanini M. Gratl V. Bettinelli P. Nicolini D.. (2019). *R*-loci arrangement versus downy and powdery mildew resistance level: A *Vitis* hybrid survey. Int. J. Mol. Sci. 18;20 (14), 3526. doi: 10.3390/ijms20143526 PMC6679420 31323823

