# Supplementary figures and images for: Secondary and primary metabolites reveal putative resistance-associated biomarkers against Erysiphe necator in resistant grapevine genotypes
Source: Front Plant Sci. 2023 Jan 31;14:1112157. doi: 10.3389/fpls.2023.1112157 (PMC9927228; doi:10.3389/fpls.2023.1112157)

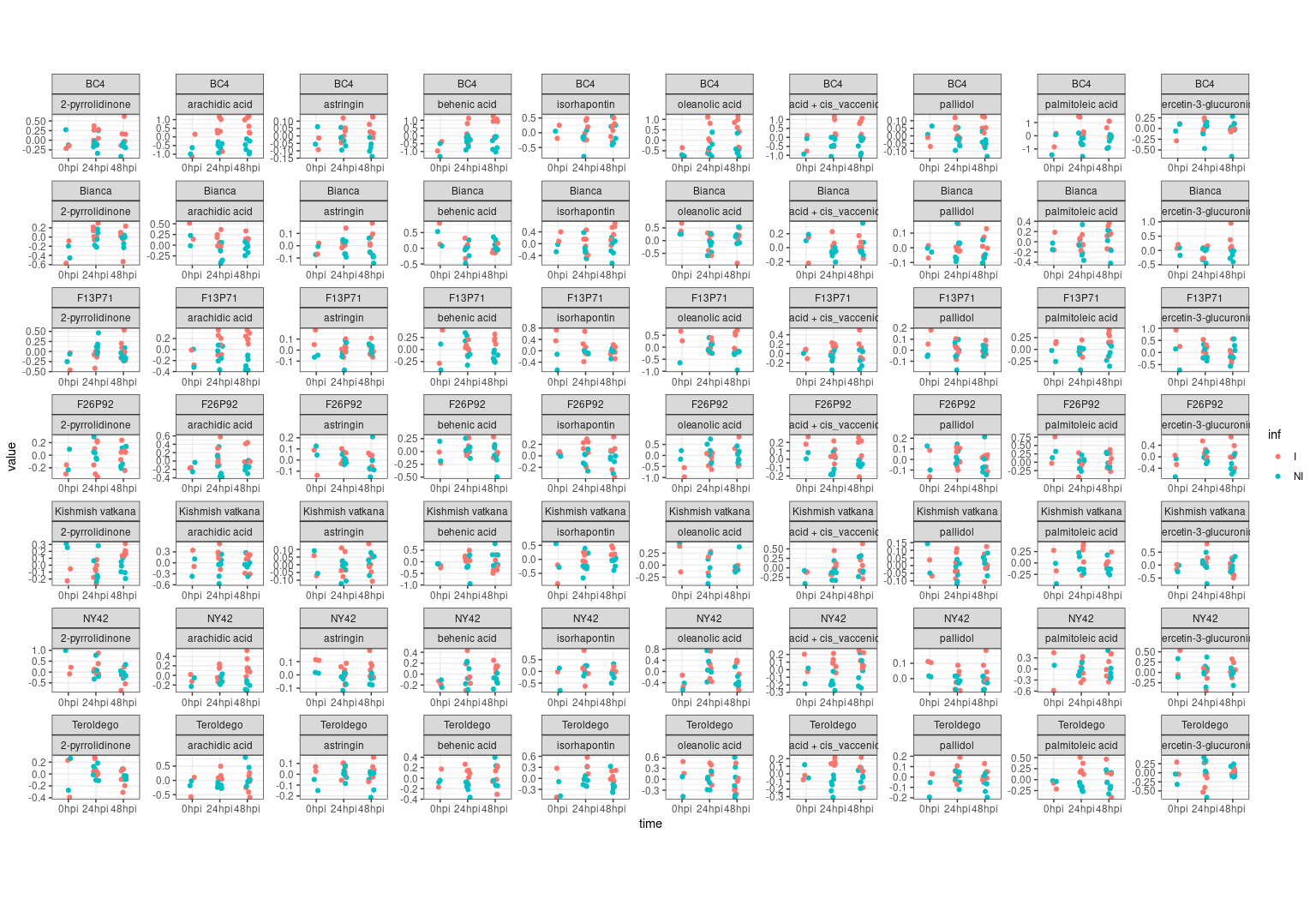

Supplement: Supplementary file 7 [file Image_1.png]
