# Supplementary material for: Secondary and primary metabolites reveal putative resistance-associated biomarkers against Erysiphe necator in resistant grapevine genotypes
Source: Front Plant Sci. 2023 Jan 31;14:1112157. doi: 10.3389/fpls.2023.1112157 (PMC9927228; doi:10.3389/fpls.2023.1112157)
Supplement: Supplementary file 8 [file Presentation_1.pptx]

## Slide 1
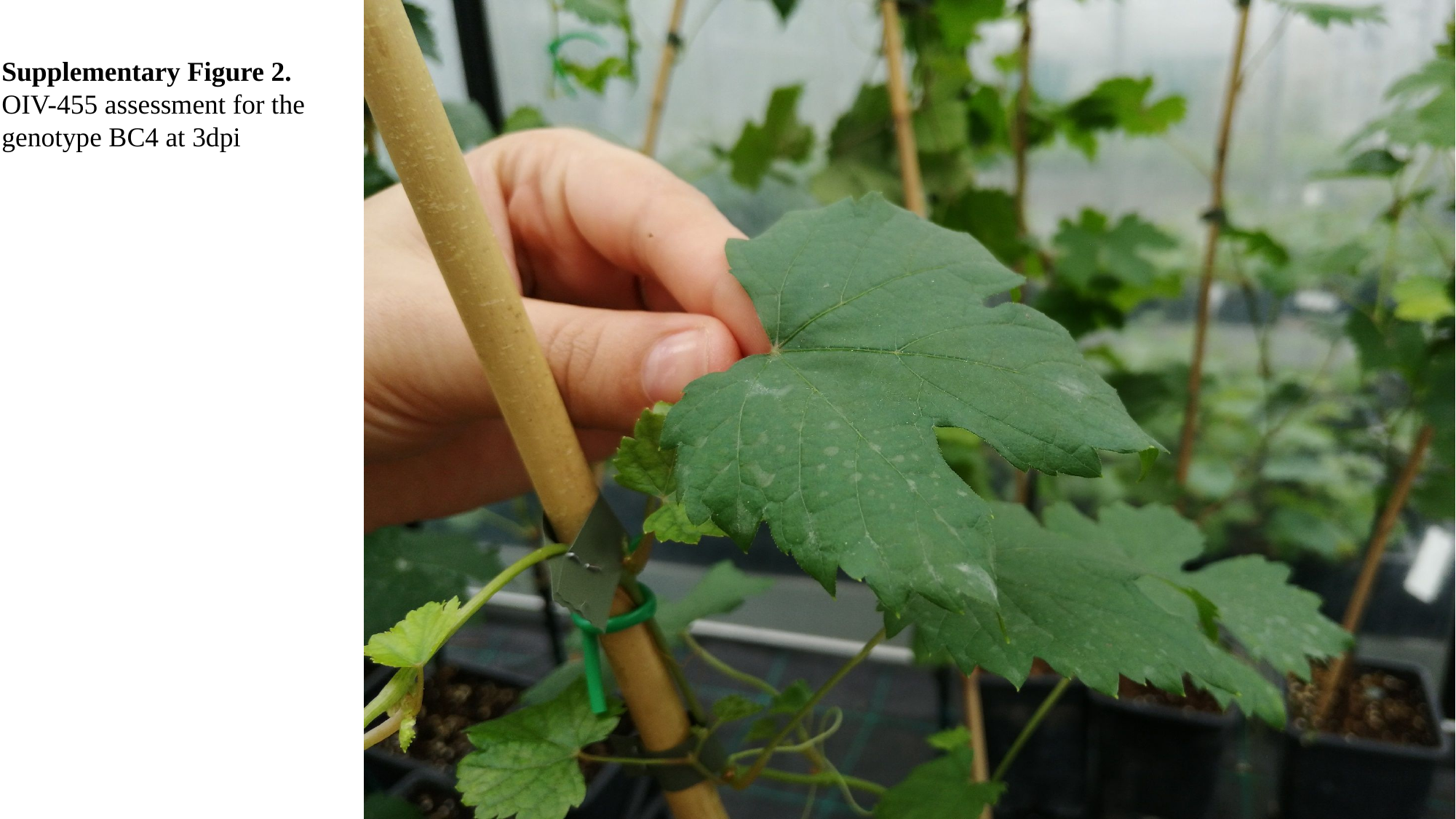

Supplementary Figure 2. OIV-455 assessment for the genotype BC4 at 3dpi

## Slide 2
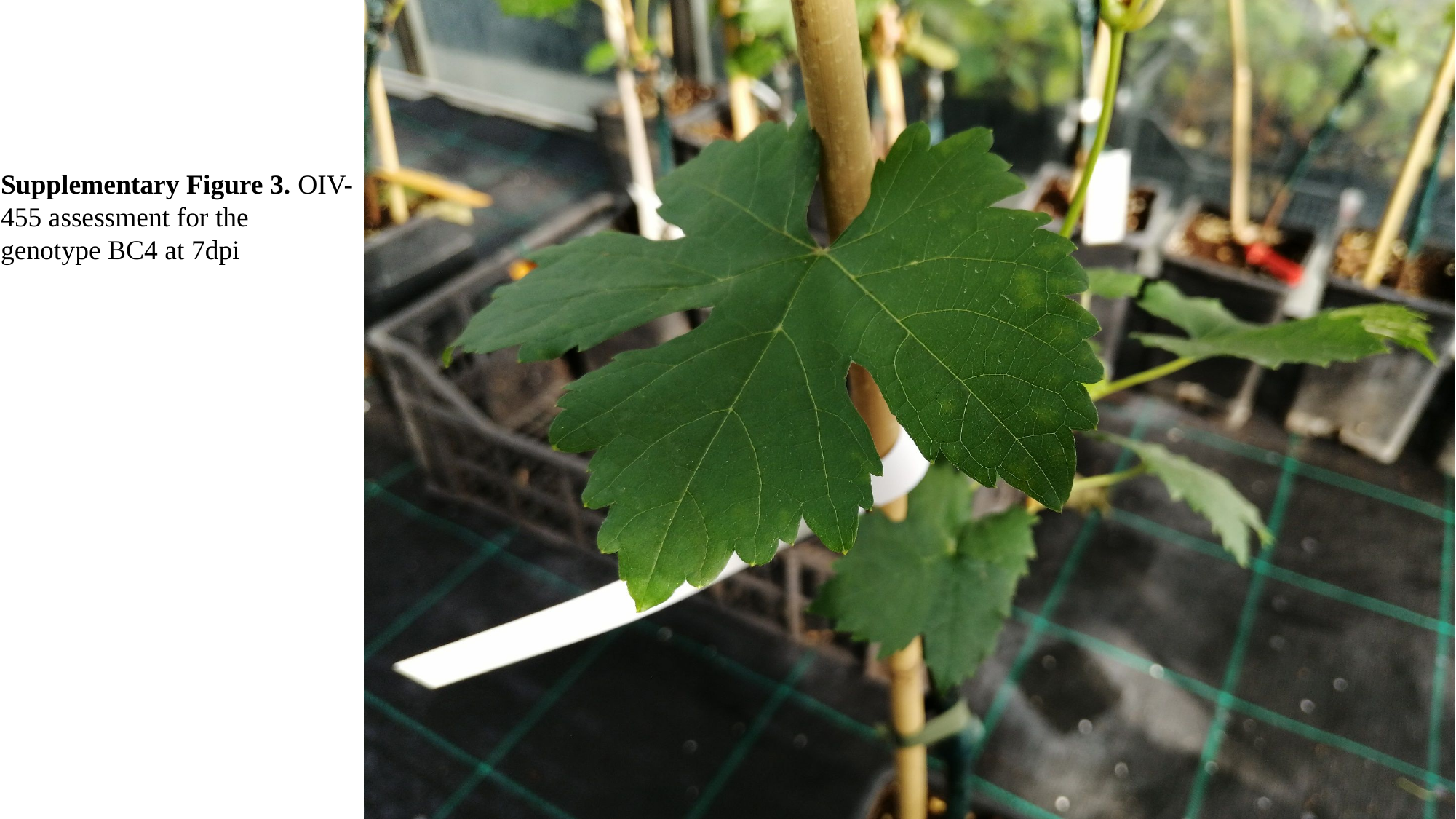

Supplementary Figure 3. OIV-455 assessment for the genotype BC4 at 7dpi

## Slide 3
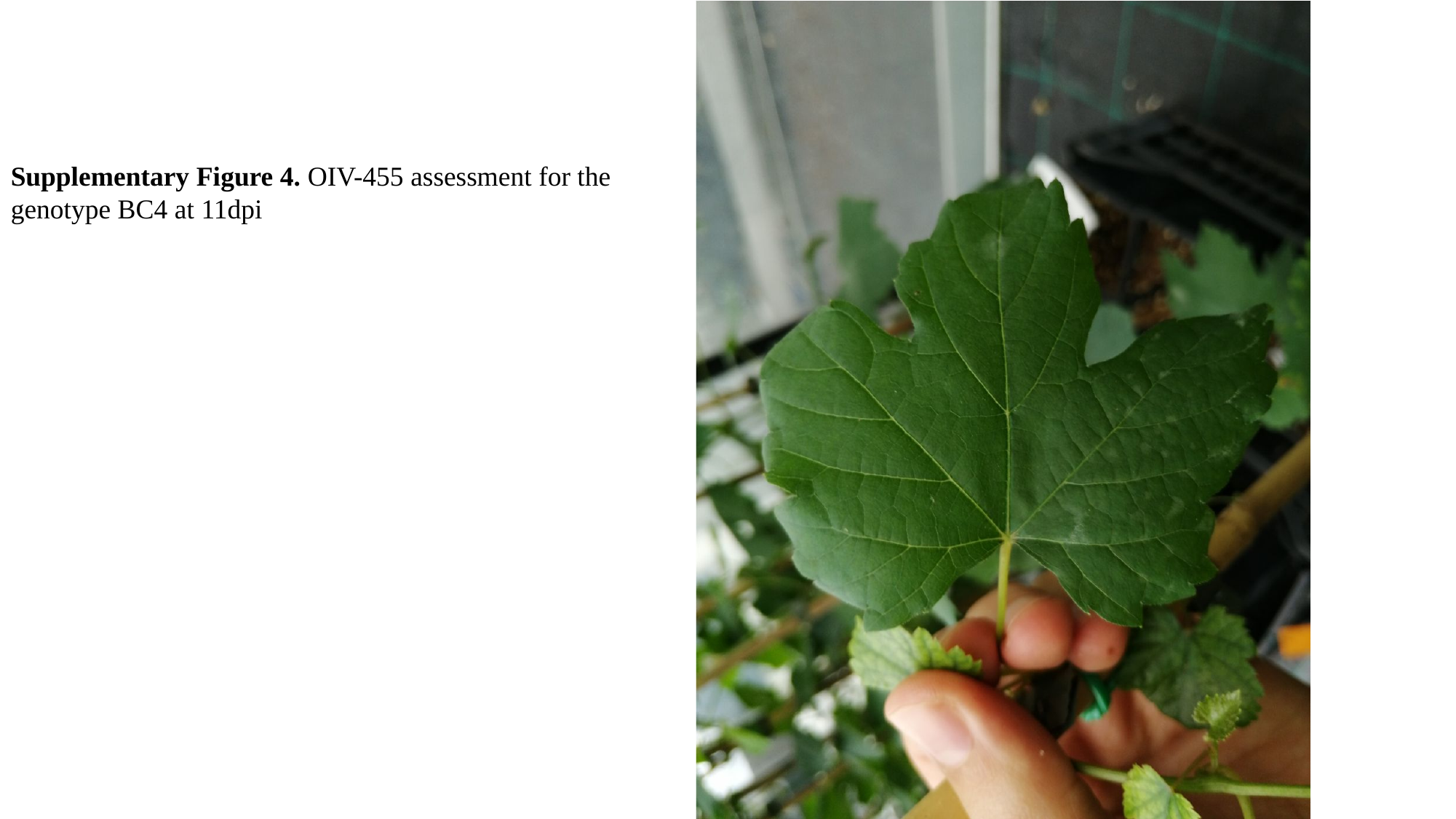

Supplementary Figure 4. OIV-455 assessment for the genotype BC4 at 11dpi

## Slide 4
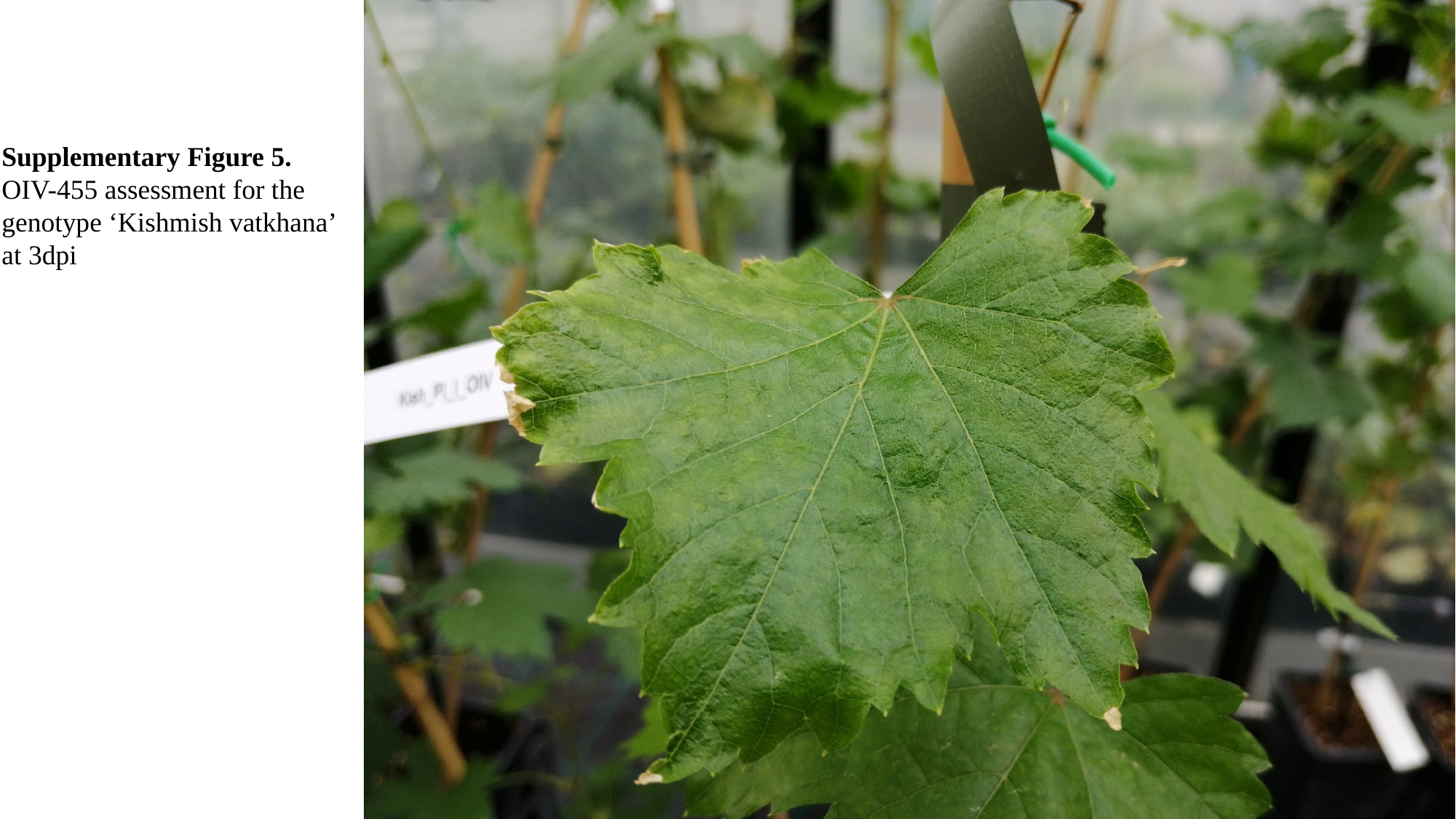

Supplementary Figure 5. OIV-455 assessment for the genotype ‘Kishmish vatkhana’ at 3dpi

## Slide 5
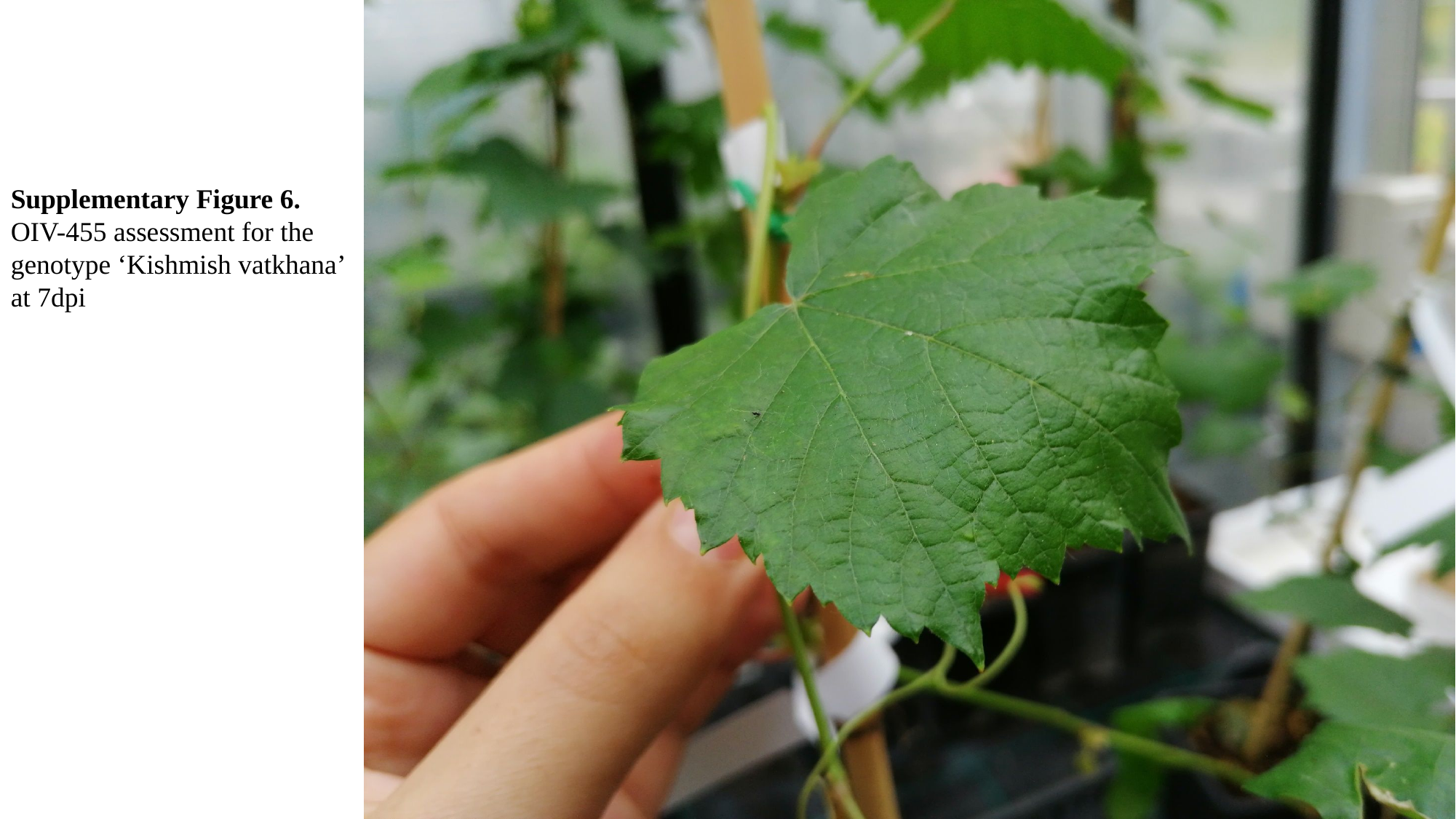

Supplementary Figure 6. OIV-455 assessment for the genotype ‘Kishmish vatkhana’ at 7dpi

## Slide 6
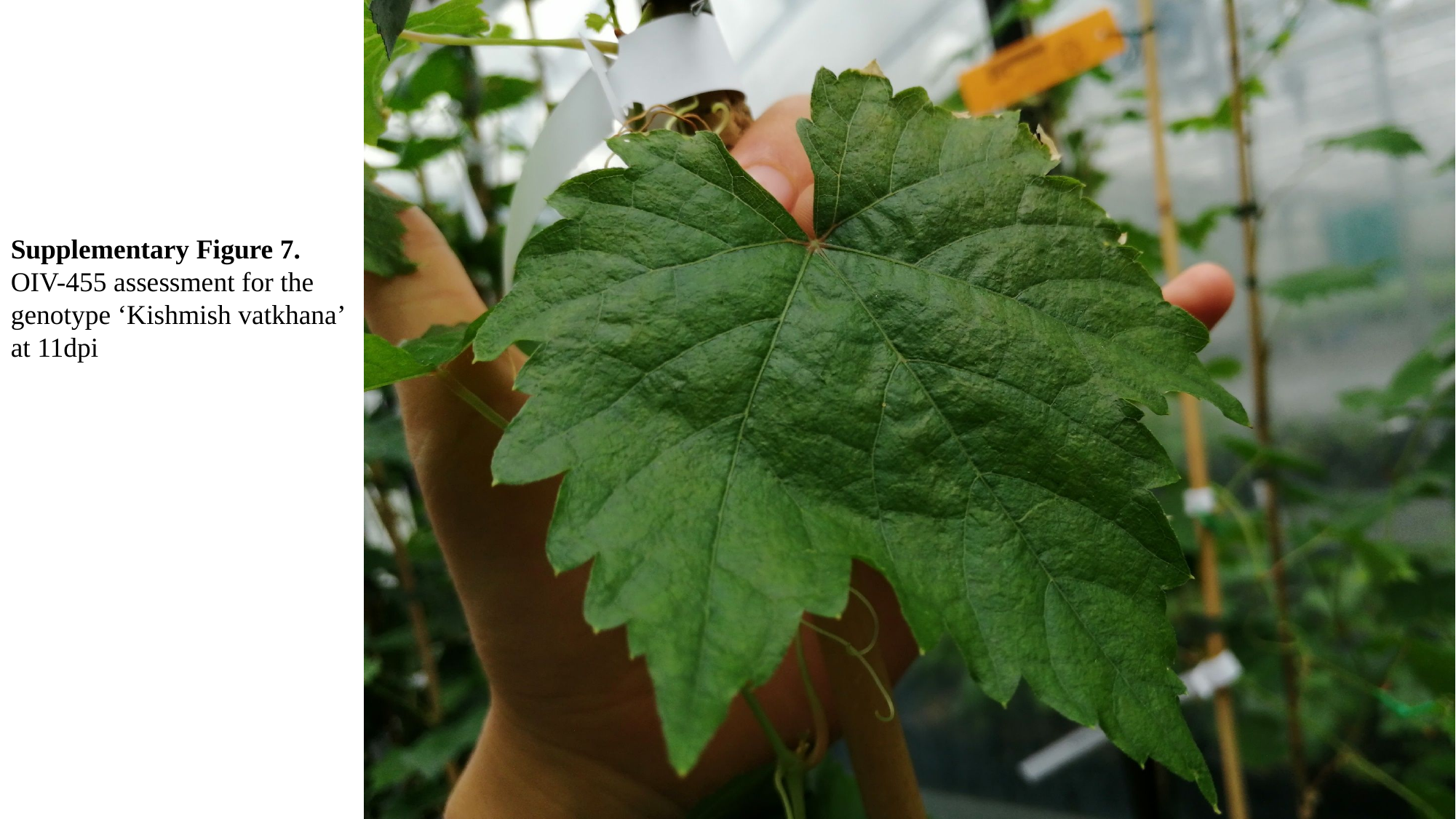

Supplementary Figure 7. OIV-455 assessment for the genotype ‘Kishmish vatkhana’ at 11dpi

## Slide 7
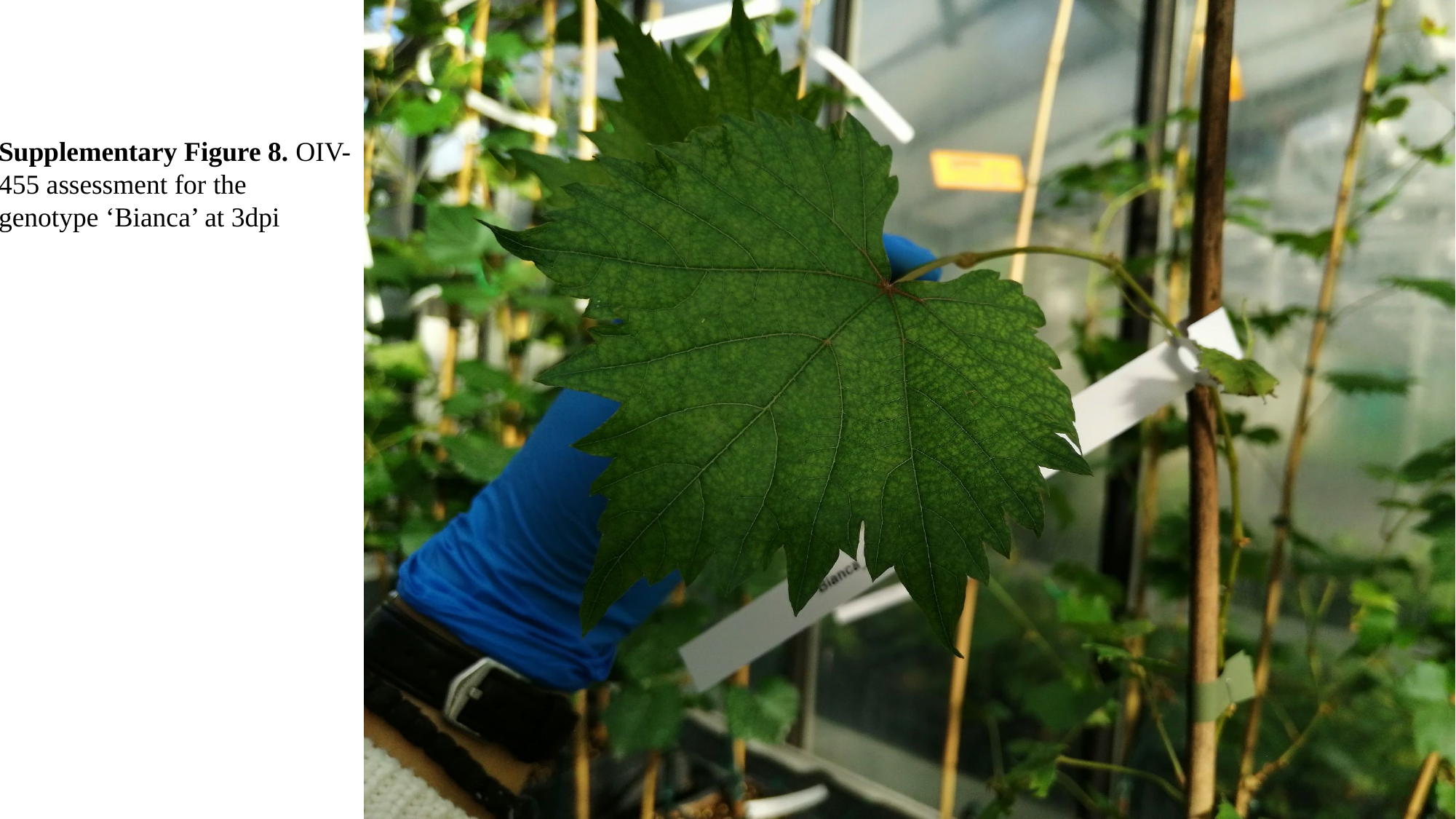

Supplementary Figure 8. OIV-455 assessment for the genotype ‘Bianca’ at 3dpi

## Slide 8
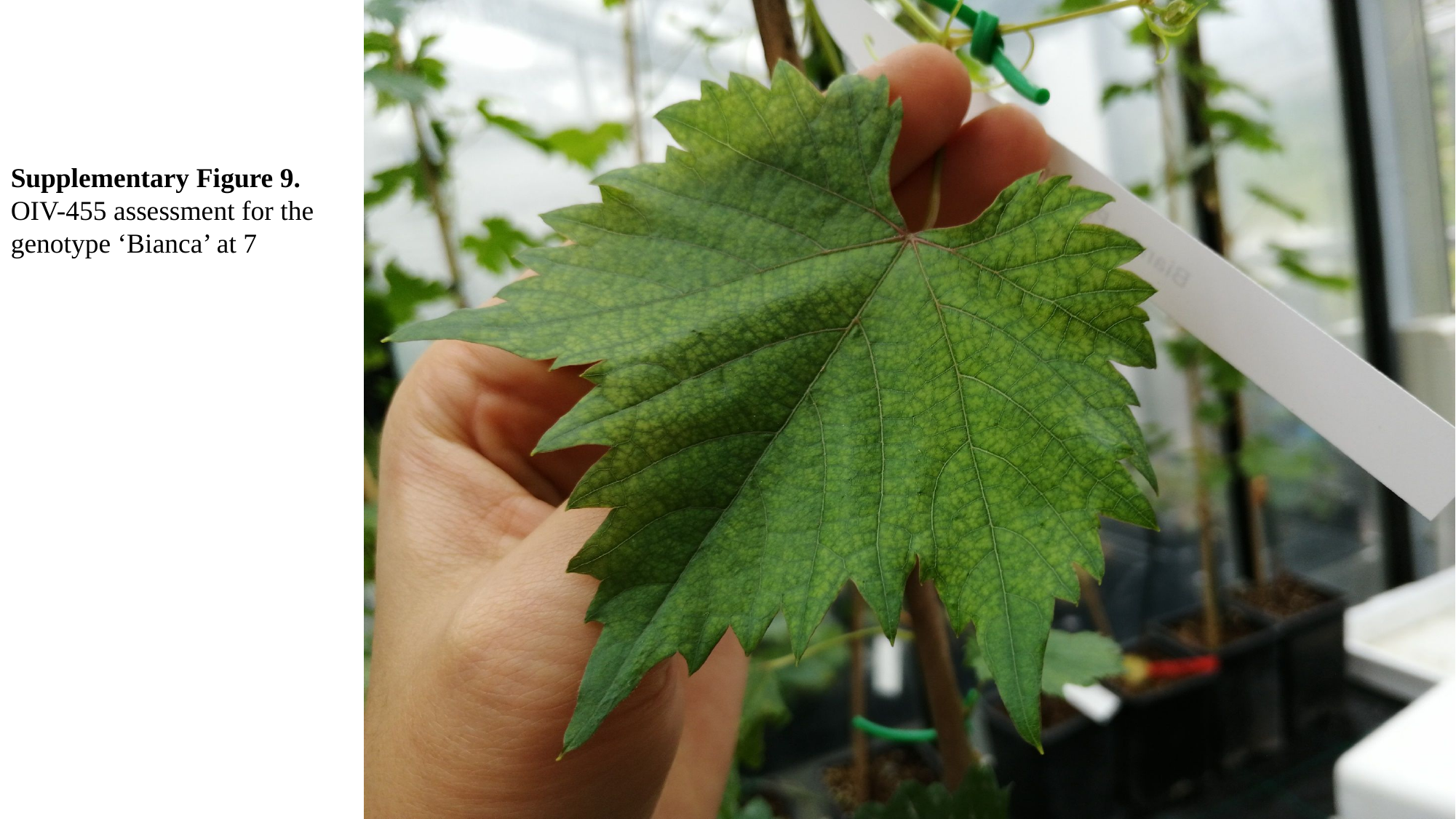

Supplementary Figure 9. OIV-455 assessment for the genotype ‘Bianca’ at 7

## Slide 9
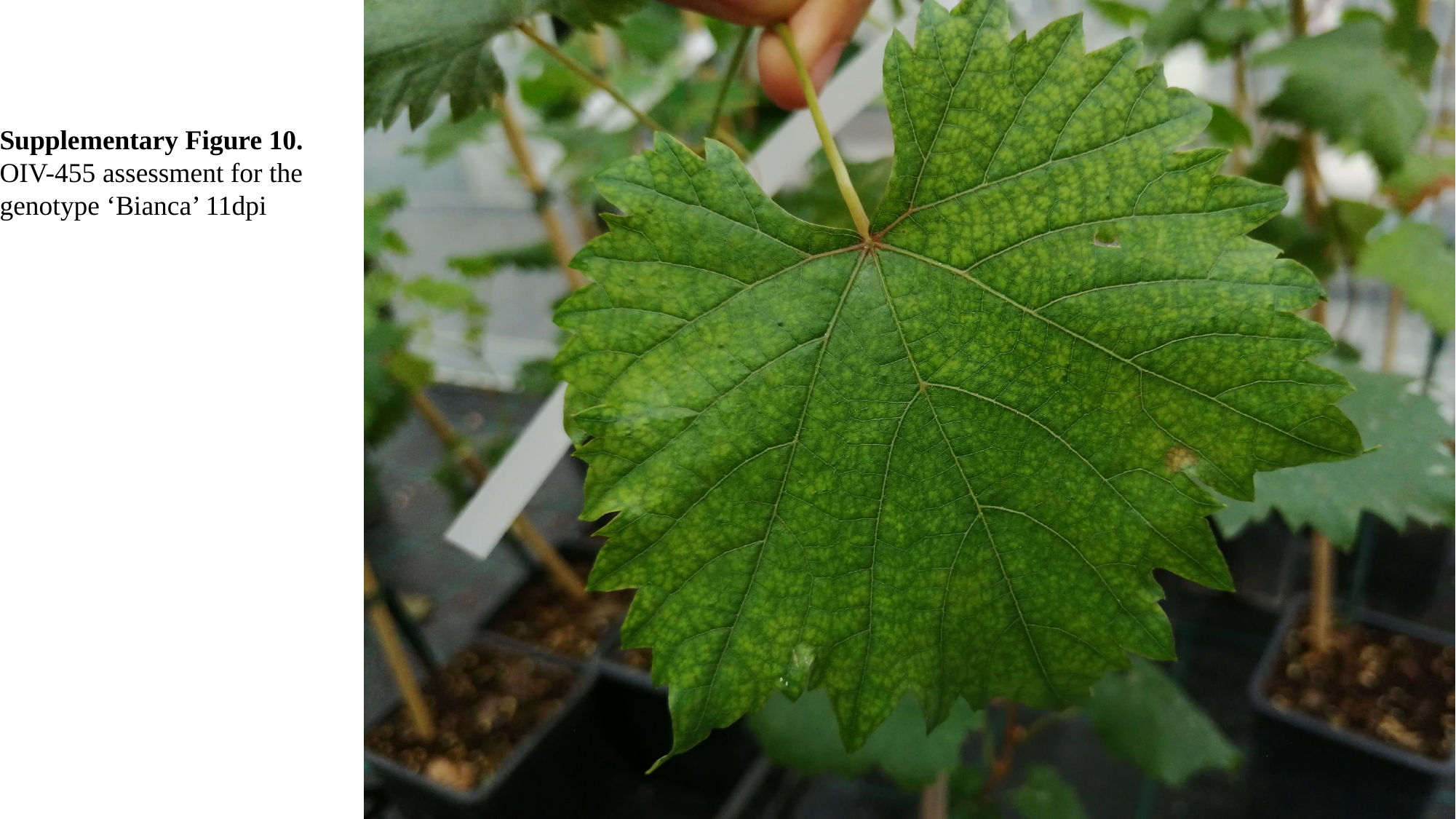

Supplementary Figure 10. OIV-455 assessment for the genotype ‘Bianca’ 11dpi

## Slide 10
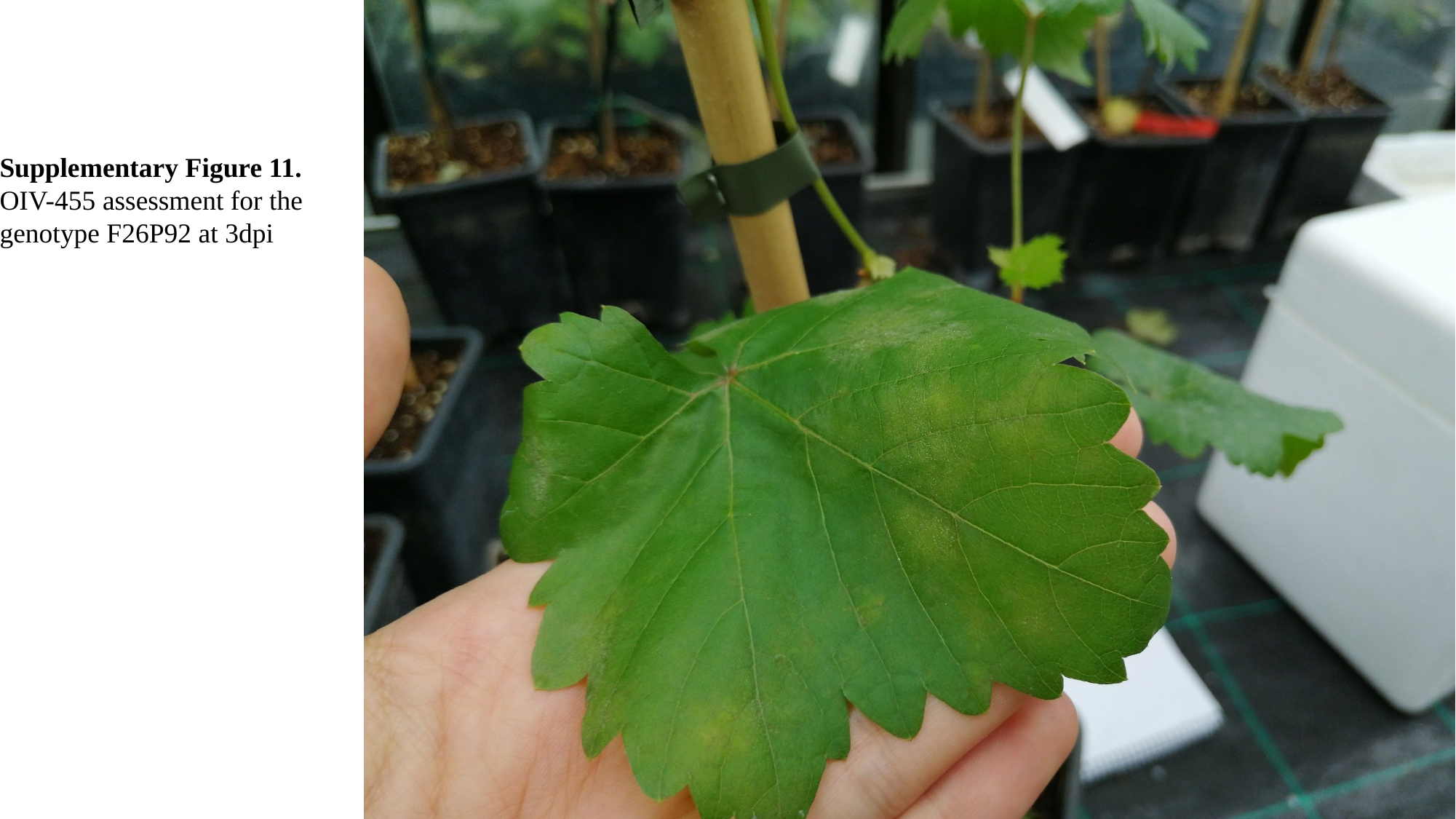

Supplementary Figure 11. OIV-455 assessment for the genotype F26P92 at 3dpi

## Slide 11
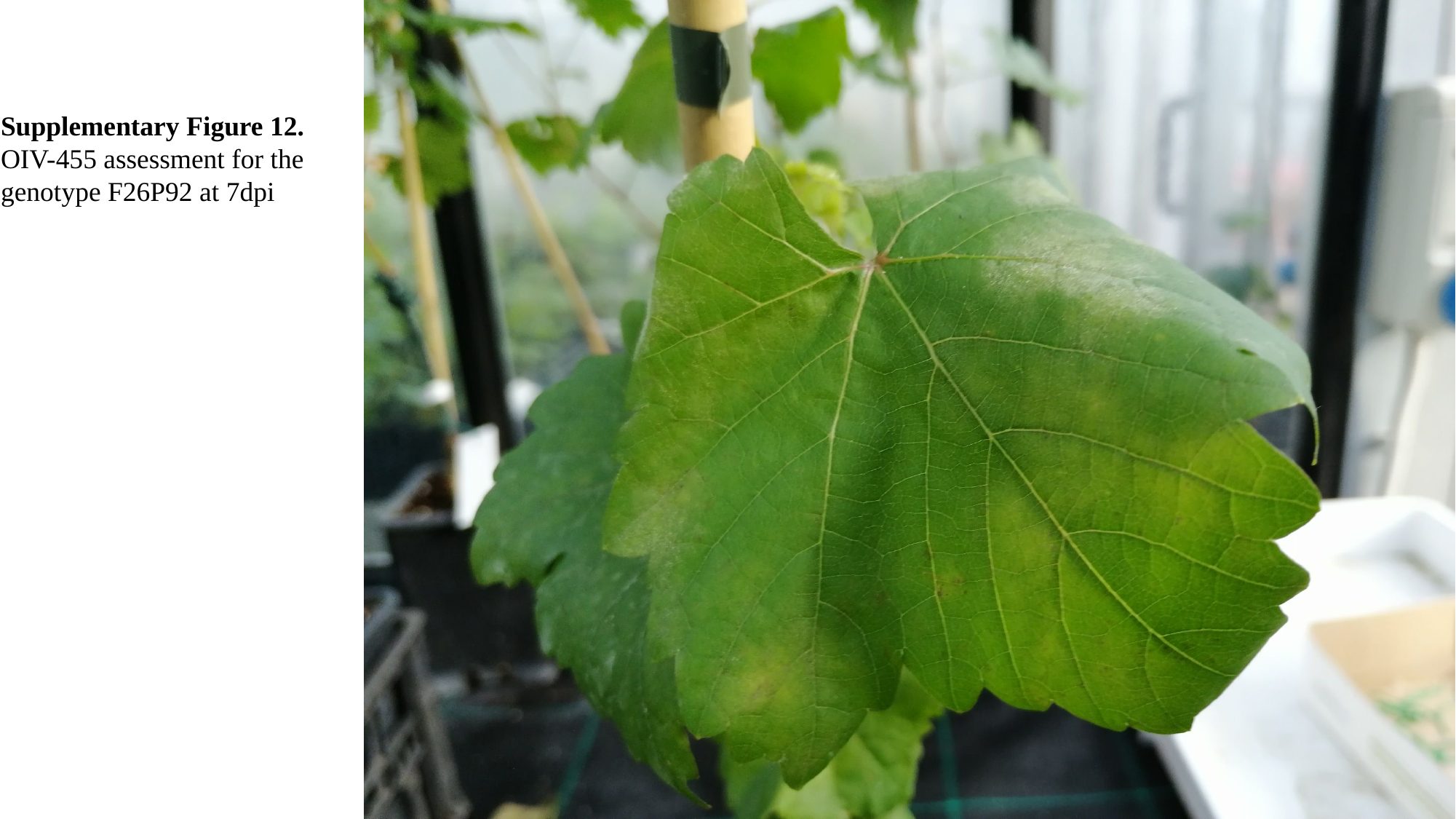

Supplementary Figure 12. OIV-455 assessment for the genotype F26P92 at 7dpi

## Slide 12
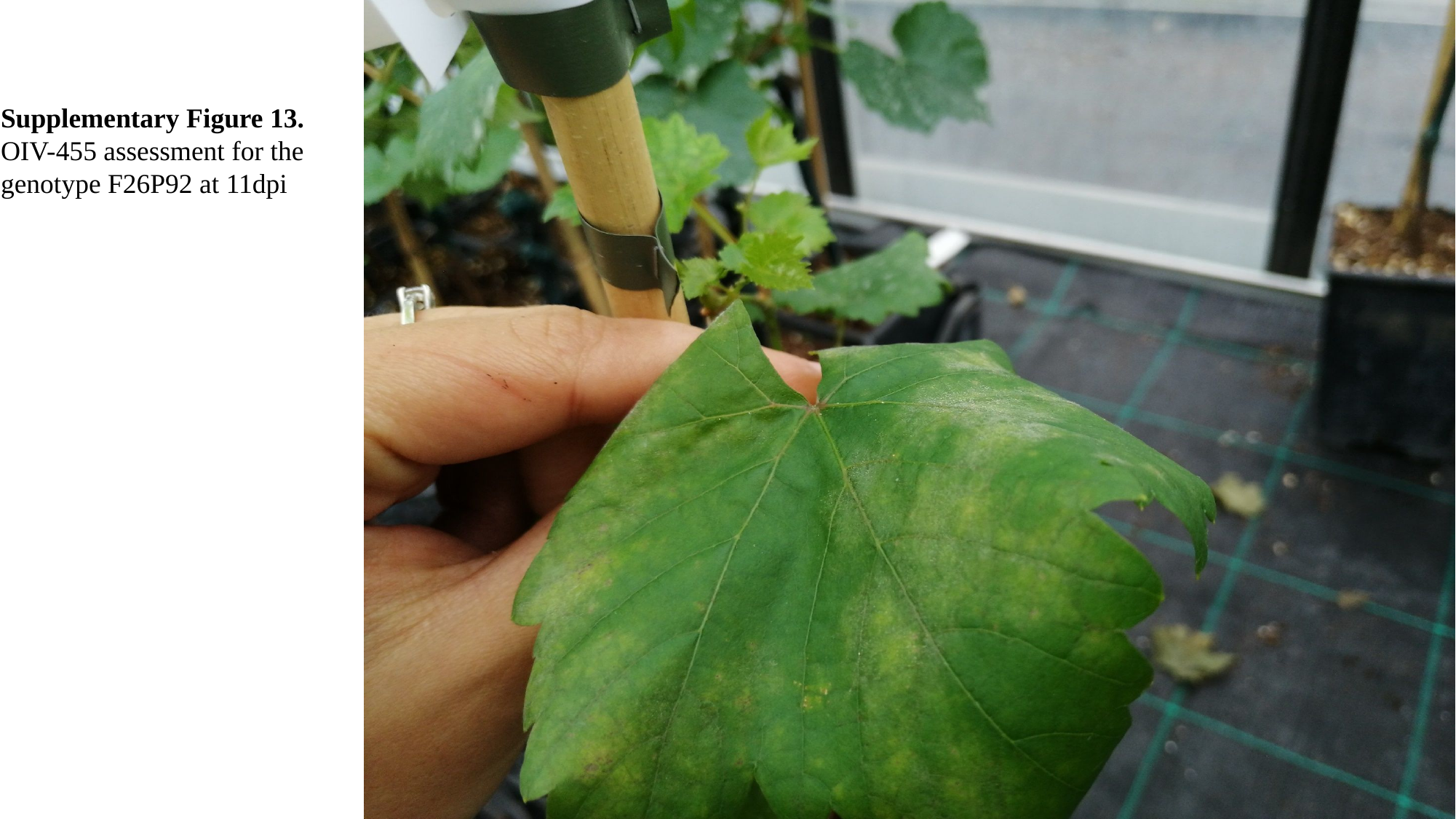

Supplementary Figure 13. OIV-455 assessment for the genotype F26P92 at 11dpi

## Slide 13
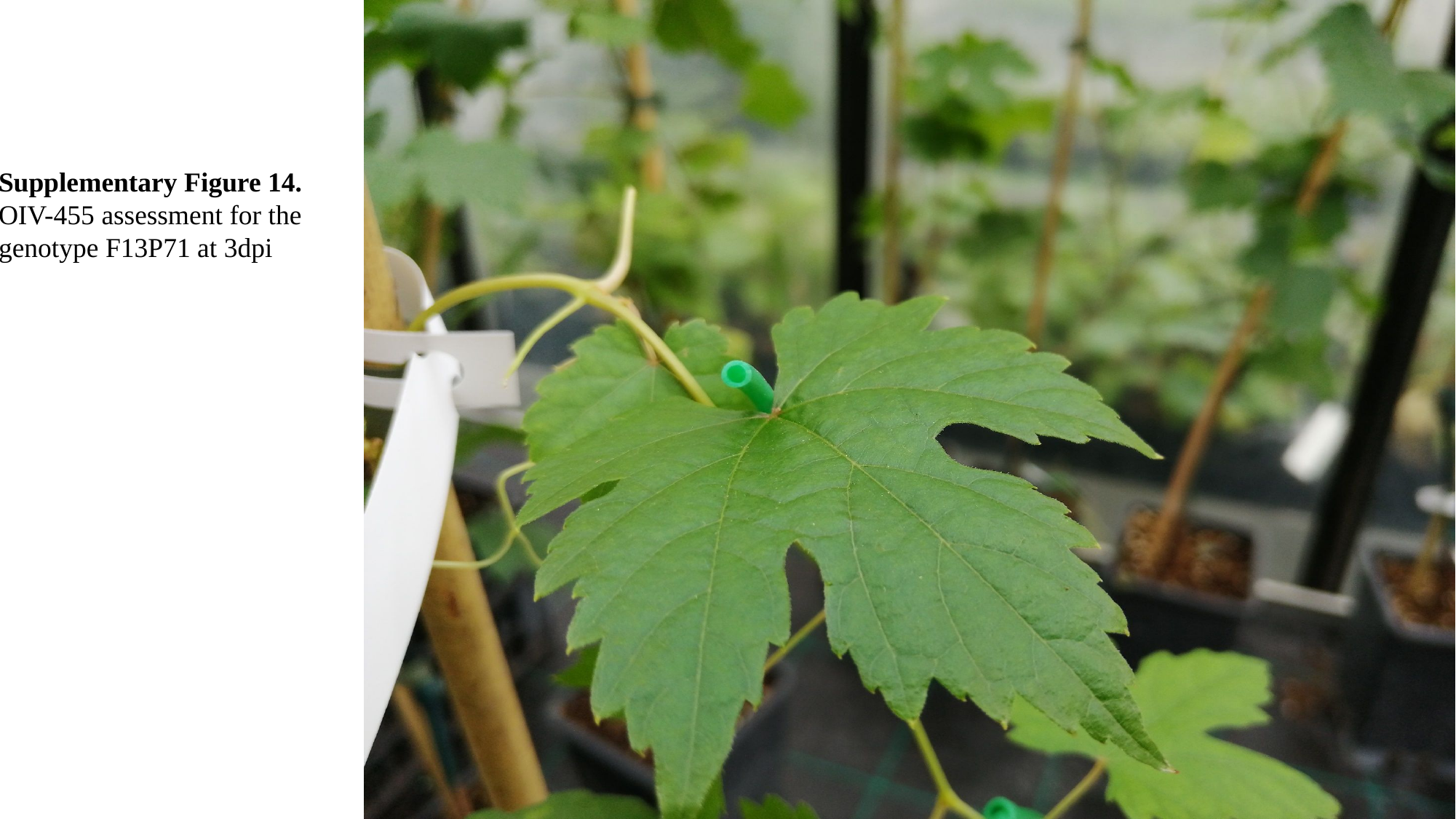

Supplementary Figure 14. OIV-455 assessment for the genotype F13P71 at 3dpi

## Slide 14
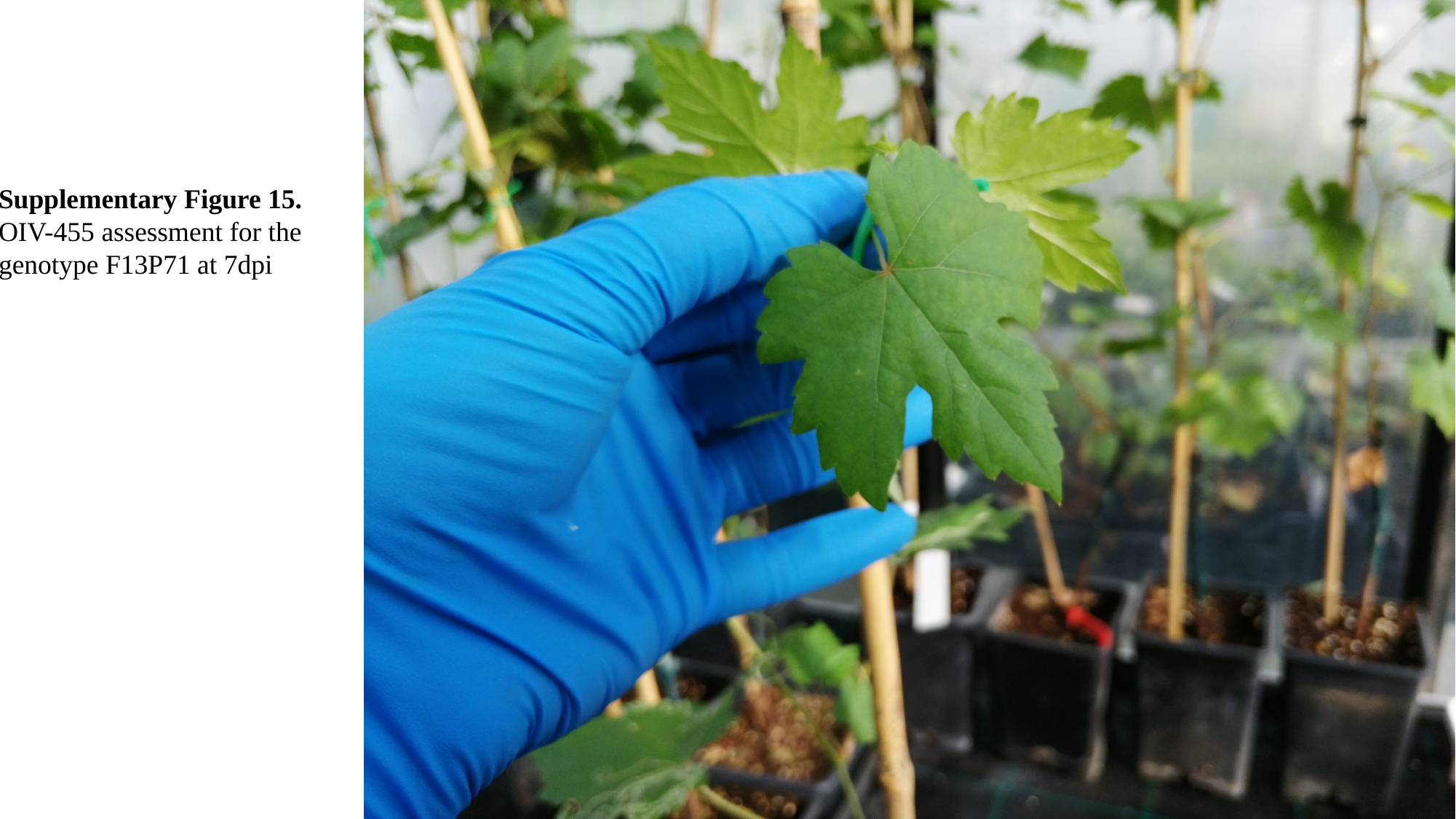

Supplementary Figure 15. OIV-455 assessment for the genotype F13P71 at 7dpi

## Slide 15
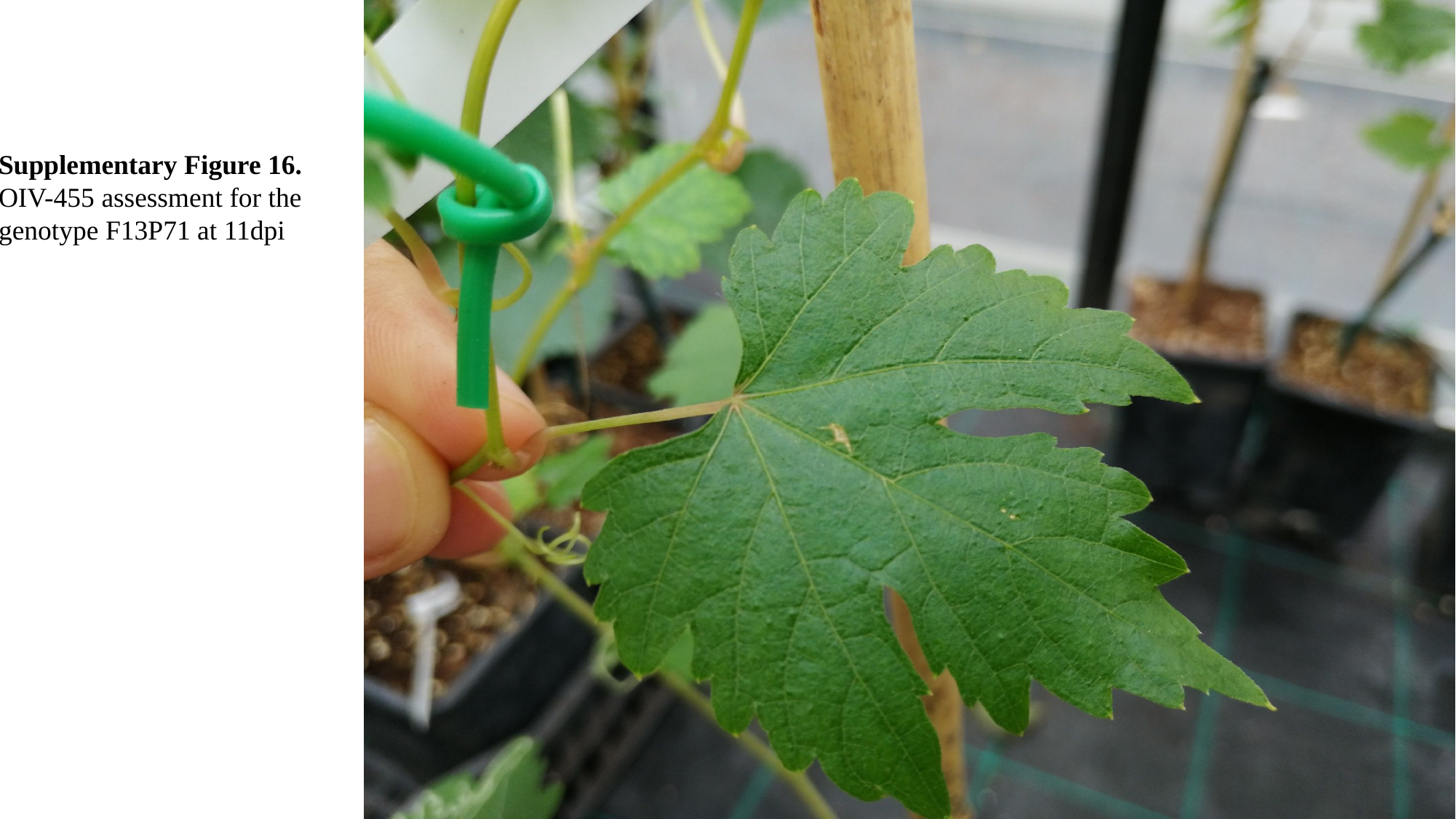

Supplementary Figure 16. OIV-455 assessment for the genotype F13P71 at 11dpi

## Slide 16
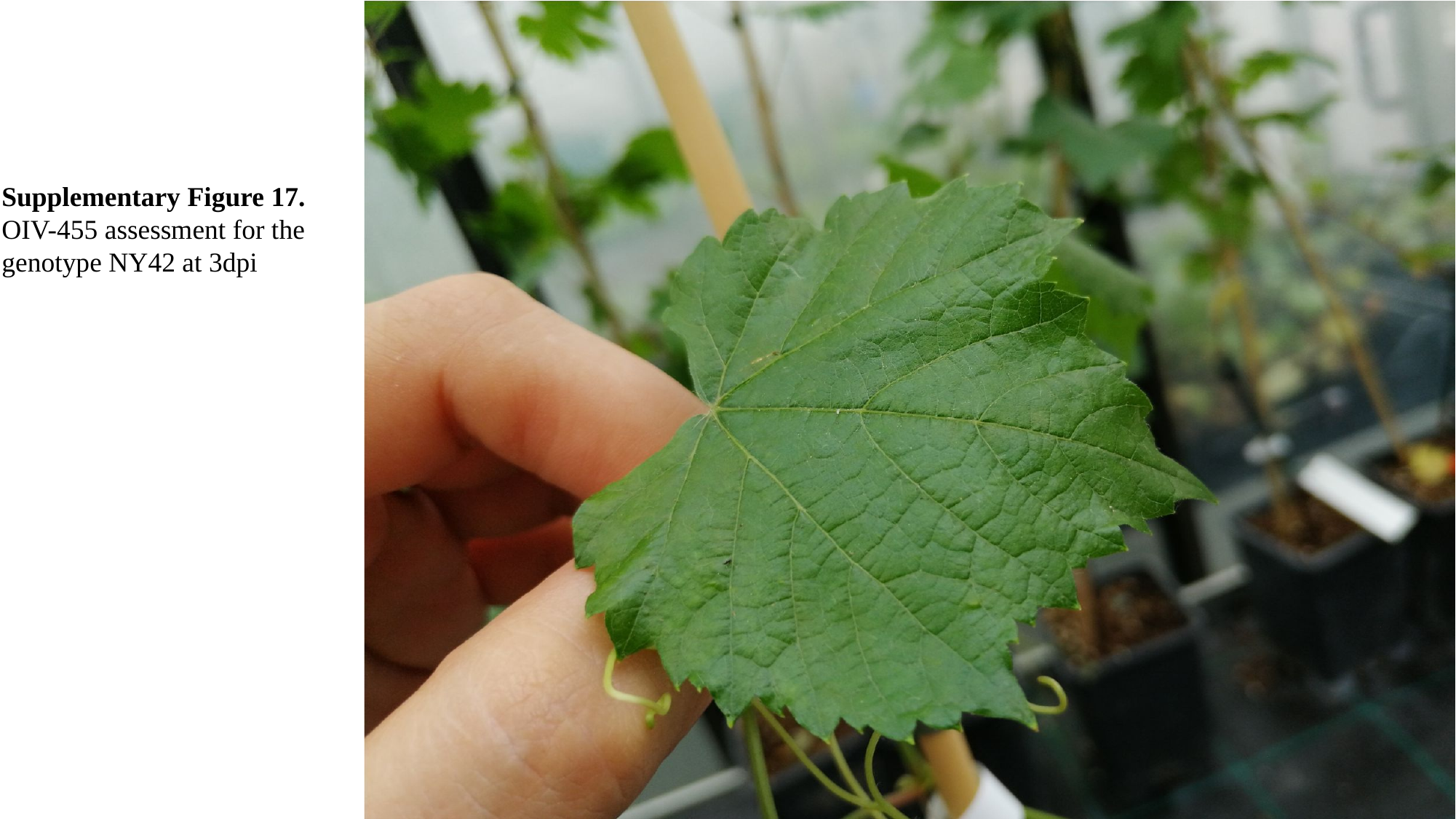

Supplementary Figure 17. OIV-455 assessment for the genotype NY42 at 3dpi

## Slide 17
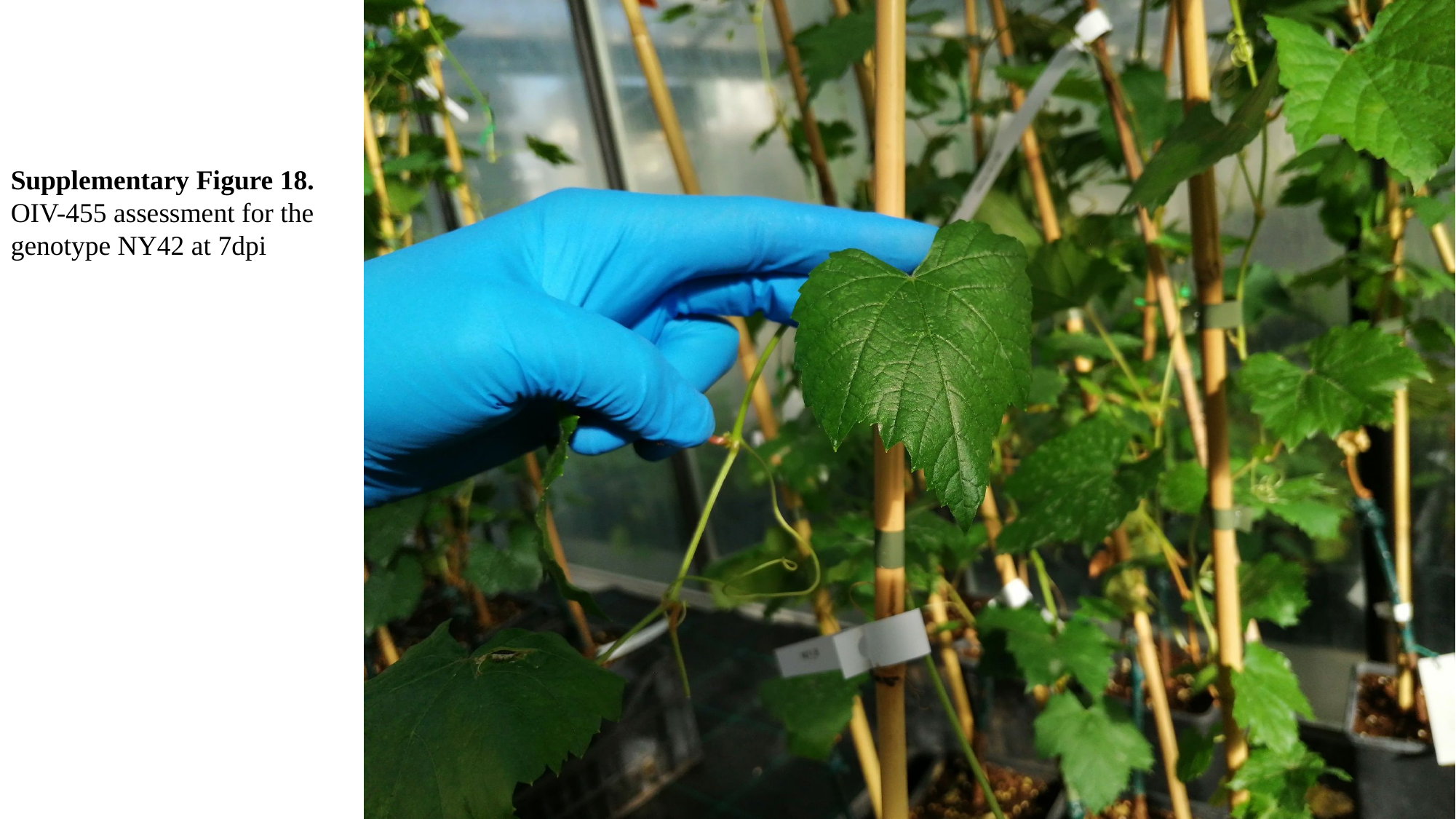

Supplementary Figure 18. OIV-455 assessment for the genotype NY42 at 7dpi

## Slide 18
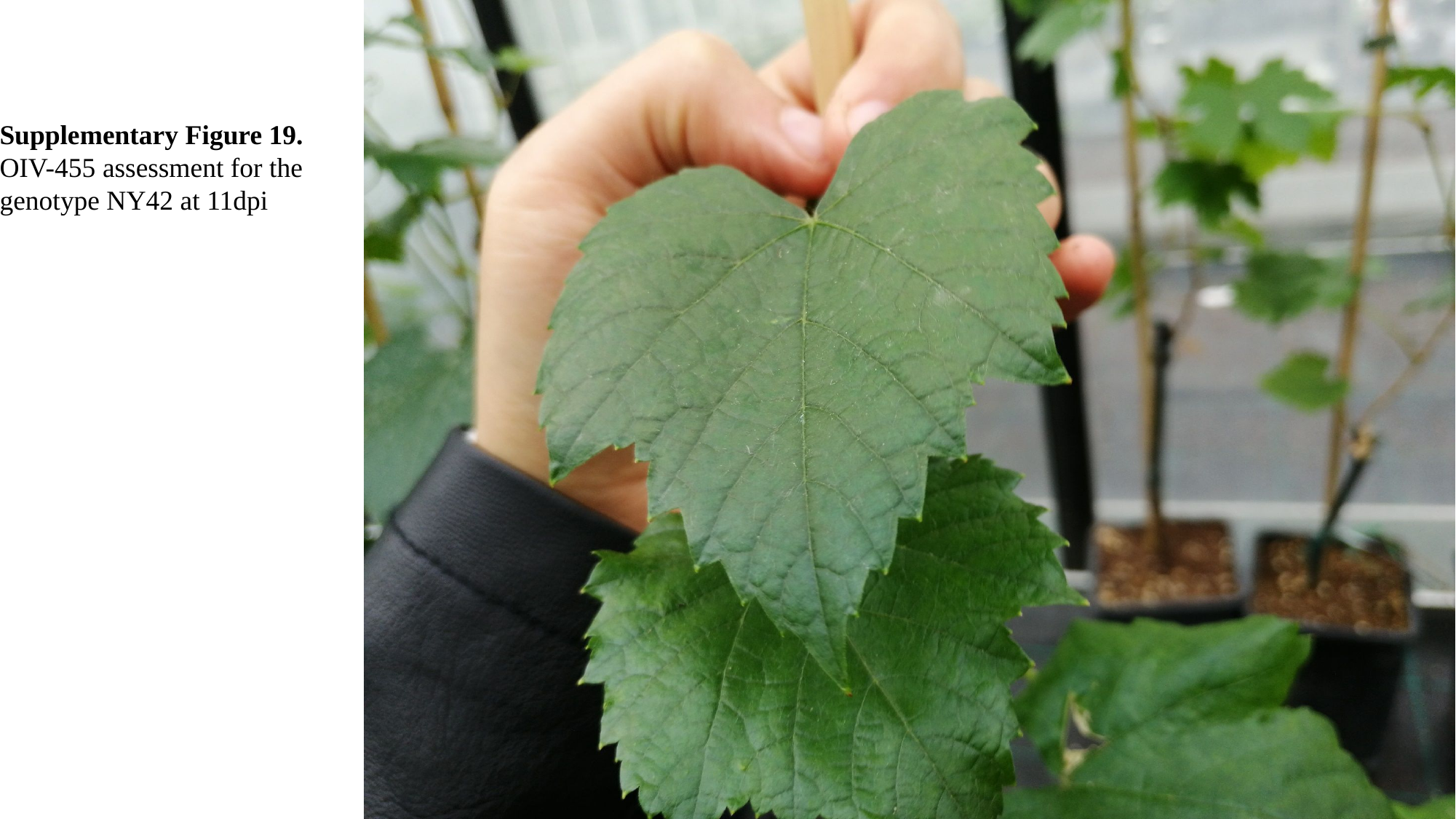

Supplementary Figure 19. OIV-455 assessment for the genotype NY42 at 11dpi

## Slide 19
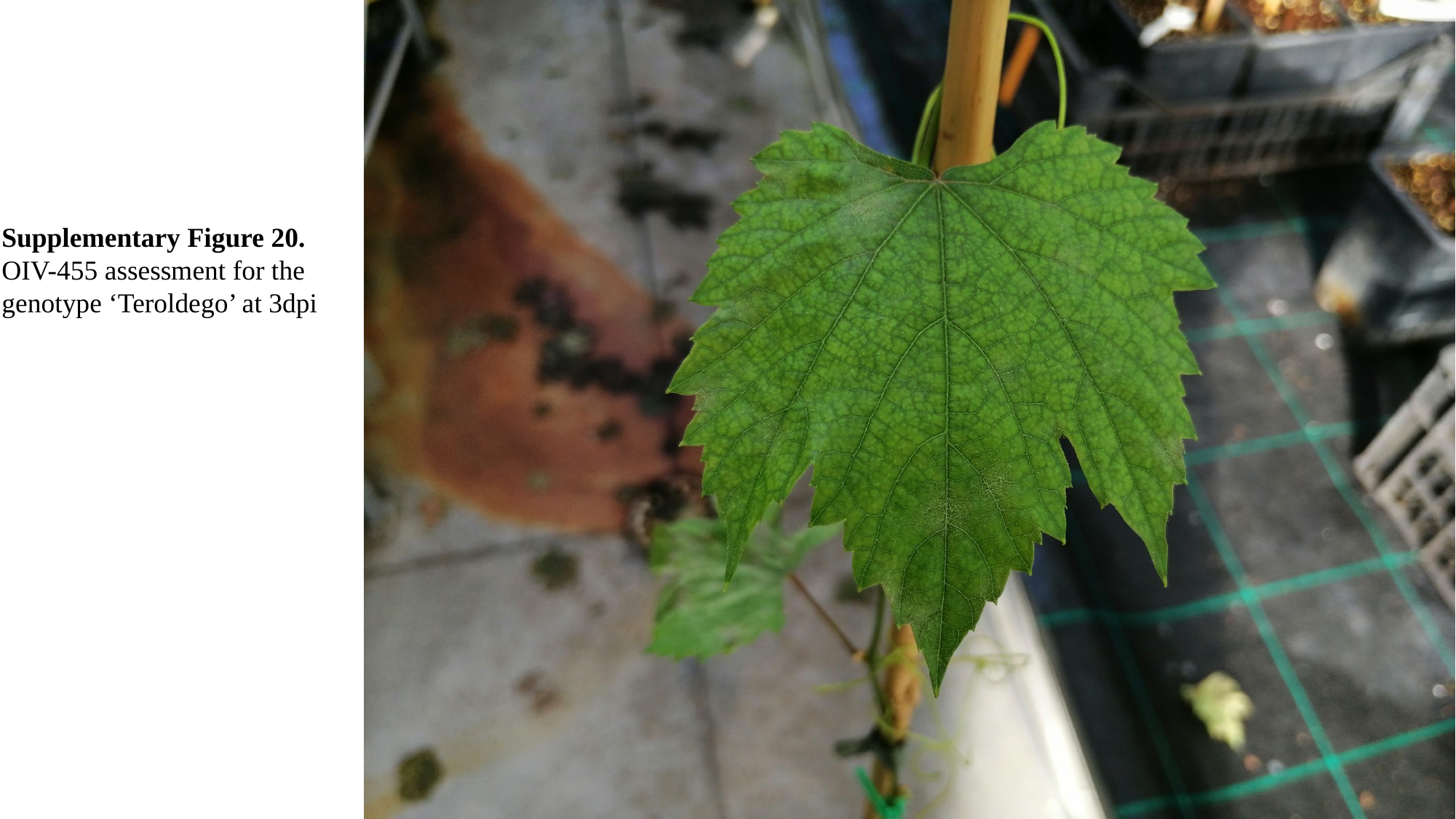

Supplementary Figure 20. OIV-455 assessment for the genotype ‘Teroldego’ at 3dpi

## Slide 20
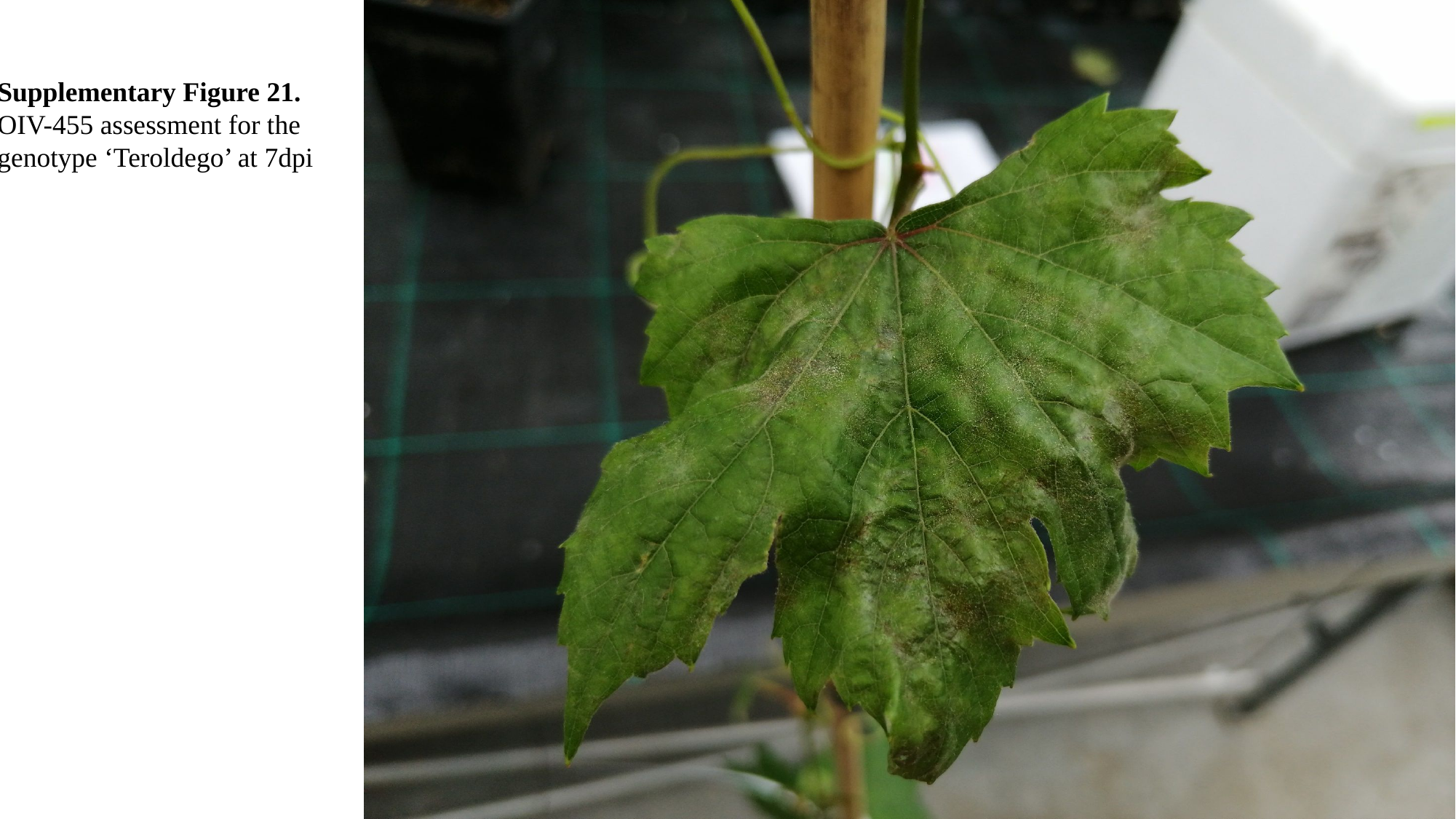

Supplementary Figure 21. OIV-455 assessment for the genotype ‘Teroldego’ at 7dpi

## Slide 21
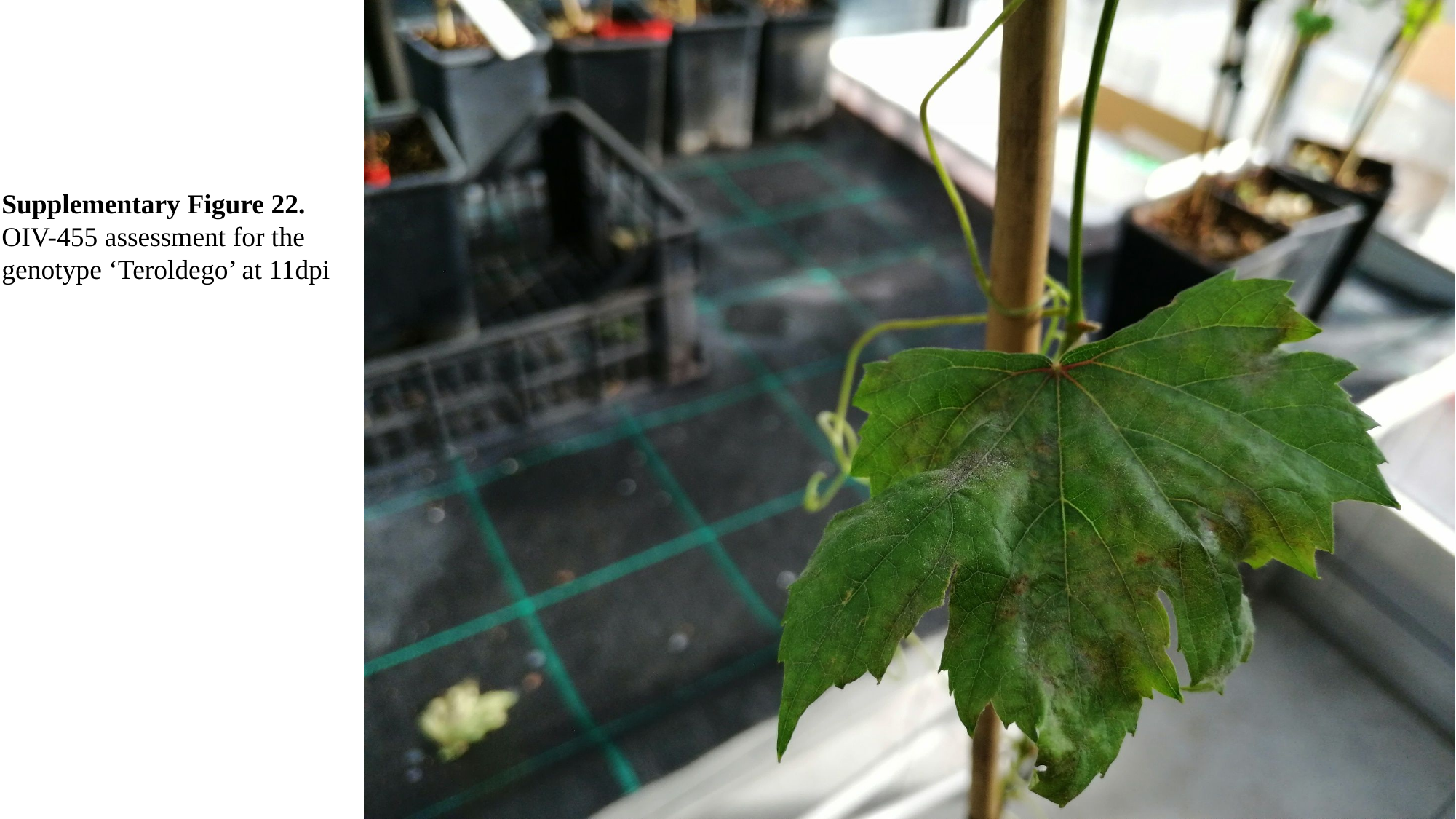

Supplementary Figure 22. OIV-455 assessment for the genotype ‘Teroldego’ at 11dpi
